# ^19^F‐NMR Reveals the Role of Mobile Loops in Product and Inhibitor Binding by the São Paulo Metallo‐β‐Lactamase

**DOI:** 10.1002/anie.201612185

**Published:** 2017-03-02

**Authors:** Martine I. Abboud, Philip Hinchliffe, Jürgen Brem, Robert Macsics, Inga Pfeffer, Anne Makena, Klaus‐Daniel Umland, Anna M. Rydzik, Guo‐Bo Li, James Spencer, Timothy D. W. Claridge, Christopher J. Schofield

**Affiliations:** ^1^ Department of ChemistryUniversity of Oxford12 Mansfield RoadOX1 3TAOxfordUK; ^2^ School of Cellular and Molecular MedicineUniversity of BristolBristolUK

**Keywords:** antibiotic resistance, β-lactamases, NMR spectroscopy, protein structures, São Paulo metallo-β-lactamase

## Abstract

Resistance to β‐lactam antibiotics mediated by metallo‐β‐lactamases (MBLs) is a growing problem. We describe the use of protein‐observe ^19^F‐NMR (PrOF NMR) to study the dynamics of the São Paulo MBL (SPM‐1) from β‐lactam‐resistant *Pseudomonas aeruginosa*. Cysteinyl variants on the α3 and L3 regions, which flank the di‐Zn^II^ active site, were selectively ^19^F‐labeled using 3‐bromo‐1,1,1‐trifluoroacetone. The PrOF NMR results reveal roles for the mobile α3 and L3 regions in the binding of both inhibitors and hydrolyzed β‐lactam products to SPM‐1. These results have implications for the mechanisms and inhibition of MBLs by β‐lactams and non‐β‐lactams and illustrate the utility of PrOF NMR for efficiently analyzing metal chelation, identifying new binding modes, and studying protein binding from a mixture of equilibrating isomers.

Hydrolysis catalyzed by β‐lactamases is one of the most important mechanisms of resistance to β‐lactam antibiotics.^1^ Although β‐lactamases employing a mechanism involving a nucleophilic serine (classes A, C, and D) have well‐established roles in resistance to β‐lactams, the class B Zn^II^‐dependent metallo‐β‐lactamases (MBLs) have more recently emerged as a major clinical problem (Figure 1 A).^2^ Clinically useful inhibitors of the class A β‐lactamases (e.g., clavulanic acid) are widely used, and avibactam has recently been reported as a broad‐spectrum serine β‐lactamase inhibitor;^3^ however, no such inhibitors exist for the MBLs.^4^

**Figure 1:**
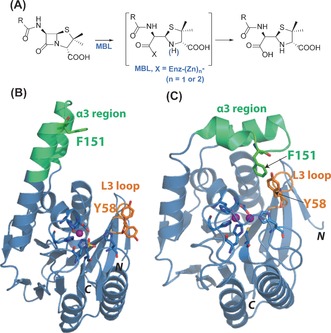
A) Outline mechanism for metallo‐β‐lactamases (MBLs). Views of SPM‐1 structures in B) “open” (PDB ID: 2FHX)8a and C) “closed” (PDB ID: 4BP0)8b conformations of the α3 region. (Y58 was refined in two conformations in the former).8a SPM‐1 has a characteristic elongated α3 region (green) and a short L3 loop (orange). Sites of labeling by cysteine alkylation with 3‐bromo‐1,1,1‐trifluoroacteone are identified by residue numbers. Note that the active cysteine (Cys221) is not labeled since it chelates Zn^II^.

The São Paulo MBL‐1 (SPM‐1) was first identified in β‐lactam‐resistant *Pseudomonas aeruginosa*,^5^ and SPM‐1‐producing *P. aeruginosa* is endemic in Brazilian hospitals.^6^ Recent reports of SPM‐1‐mediated resistance in Europe, Asia, and North America reveal its global spread.^7^ SPM‐1 is a particular challenge from an inhibition perspective because it has a broad substrate specificity (catalyzing penicillin, cephalosporin, and carbapenem hydrolysis) and has properties characteristic of both B1‐ and B2‐subfamily MBLs (Figure S1 in the Supporting Information).^8^ SPM‐1 resembles B1 MBLs in terms of its di‐Zn^II^ ion requirement and (based on available evidence) with respect to its kinetics.^9^ SPM‐1 has unusual second‐sphere residues,^10^ and is unique amongst B1 MBLs with respect to mobile active‐site regions; SPM‐1 has an extended “α3 region” (residues 223–241, BBL numbering) and a relatively short L3 loop (residues 61–66, BBL numbering), which are features characteristic of B2 MBLs.8a No structures of SPM‐1 complexed with substrates/inhibitors have been solved, though structures in which the α3 region adopts open8a and closed8b conformations with respect to the active site have been reported (Figure 1 B,C).

Owing to its intrinsic sensitivity, lack of resonance overlap, and advances in NMR instruments and probe design, protein‐observe ^19^F‐NMR (PrOF NMR) is of increasing utility in studying conformational changes and protein–ligand interactions.^11^ We have reported on the use of PrOF NMR to study MBL dynamics using cysteine alkylation by 3‐bromo‐1,1,1‐trifluoroacetone (BTFA) to efficiently introduce fluorine labels (Figure S2A).8b, 12 Here, we describe PrOF NMR studies on SPM‐1 that inform on the relative importance of the L3 loop and α3 region in the binding of different classes of MBL substrates/inhibitors. Importantly, they reveal that the hydrolyzed β‐amino acid products of MBL catalysis can bind to SPM‐1 in a process involving the L3 loop.

Residues in the L3 loop (Y58) and α3 region (F151) were selected for modification and labeling with ^19^F (Figure S2B). In initial work, we had labeled Y152;8b however, we selected F151 for further studies because analysis of SPM‐1 crystal structures^8^ implies that the F151 sidechain is mobile and projects closer to the active‐site zinc ions than that of Tyr152 (Figure S3). Selective labeling of Y58C and F151C SPM‐1 variants using BTFA (Y58C* and F151C*, respectively) was confirmed by intact‐protein and trypsin‐digest mass spectrometry (Figures S4–11). Notably, the naturally present cysteine (Cys221) in SPM‐1 was not observed to react with BTFA, likely because it chelates Zn^II^, as evidenced by *S*‐carbamidomethylation of Cys221, but not Cys58 and Cys151, in MS analyses of Y58C* and F151C* (Figures S8–11). The circular dichroism spectra^13^ of wildtype (wt) SPM‐1, Y58C*, and F151C* were similar (Figure S12), thus implying similar overall folds as supported by crystallographic analyses of Y58C (Figures S13,14 and Table S1). Kinetic analyses^14^ (Figure S15) implied that the introduction of the CH_2_COCF_3_ label did not substantially alter the substrate affinity, that is, similar *K*_M_ values were obtained for meropenem with wt SPM‐1 and both labeled variants. A 2.5‐fold decrease in *k*_cat_ for meropenem with both SPM‐1* variants was observed, possibly reflecting interactions involving the modified residue in enzyme–intermediate complexes. The combined biophysical and kinetic studies established that the properties of Y58C* and F151C* are sufficiently similar to those of wt SPM‐1 to justify PrOF NMR studies. Together with earlier studies on protein alkylation by BTFA,^15^ these results demonstrate that BTFA is useful for the efficient introduction of ^19^F labels through post‐translational cysteine alkylation.

The ^19^F‐NMR spectra revealed major protein‐observe peaks at −83.15 ppm (Y58C*) and −84.75 ppm (F151C*; Figure S16), thus indicating that the labeled loops/regions of the variants exist predominantly in a single conformation or, more likely, that the labeled residues are moving rapidly relative to the NMR shift timescale. The F151C* variant also displayed broad signals on either side of the sharper peak at −84.75 ppm, possibly reflecting conformational motion; however, we did not observe changes in the line width and intensity of the signal in variable‐temperature studies (277 K to 310 K). Consistent with the crystallographic evidence, solvent isotope exchange studies (Figure S17) revealed that F151C*, which lies in the exposed α3 region, is more solvent accessible than Y58C*, which is located in the less exposed L3 loop.

We then used PrOF NMR (Figure S18) to investigate the binding of representative MBL ligands to Y58C* and F151C* SPM‐1 (Table S2, see Table S3 for *K*_D_ values). Initially, we tested reported MBL inhibitors to validate use of the SPM‐1* variants for investigating ligand binding. With the zinc chelator 1,10‐*o*‐phenanthroline, new NMR peaks were observed for both Y58C* (Figure 2 A)and F151C* (Figure 2 B). These peaks are the same as those observed in the *apo*‐SPM‐1* spectra, which is consistent with the anticipated Zn^II^ extraction in solution by 1,10‐*o*‐phenanthroline; 1,10‐*o*‐phenanthroline itself was not observed to bind to *apo*‐Y58C* (Figure 2 A and Figures S19,20). These results reveal the utility of PrOF NMR in detecting metal chelation/binding in solution and/or to the protein, which is not always readily accessible through metallo‐enzyme inhibition studies. With rhodanine ML302 and thioenol ML302F,^16^ new peaks at −83.75 ppm and −84.40 ppm for Y58C* and F151C*, respectively, were observed (Figure S21). These observations are consistent with hydrolysis of ML302 to give the thioenol ML302F under the incubation conditions.^17^ l‐Captopril, which inhibits B1 MBLs but not SPM‐1 (IC_50_ >500 μm)^18^ and subclass B2 MBLs,^19^ did not manifest substantial changes in the ^19^F spectrum for either of the SPM‐1* variants (Figure S22).

**Figure 2:**
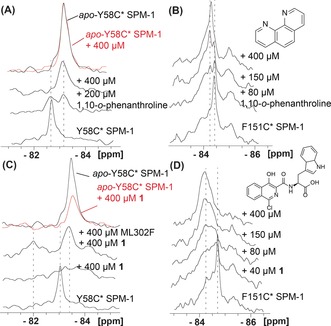
PrOF NMR monitoring of inhibitor binding to SPM‐1*. ^19^F‐NMR spectra of the interactions of 1,10‐*o*‐phenanthroline with A) Y58C* SPM‐1 and B) F151C* SPM‐1. ^19^F‐NMR spectra of the interactions of **1** with C) Y58C* SPM‐1 and D) F151C* SPM‐1. Assay mixtures: 40 μm SPM‐1* in 50 mm Tris, pH 7.5, 9:1 H_2_O/D_2_O.

Isoquinolines are broad‐spectrum MBL inhibitors,^13^, ^14^ but their binding mode is unknown. Significant line broadening, which is typical of a system in intermediate exchange, was observed when isoquinoline (**1**)^13^, ^14^ was titrated with Y58C*. Addition of ML302F^17^ to a sample containing Y58C* and **1** led to the appearance of the peak characteristic of the ML302F‐bound complex and a new peak deshielded by 1.1 ppm relative to that of the Y58C* peak (Figure 2 C). With F151C*, **1** induced broadening and chemical‐shift changes (Figure 2 D). Thus, binding of **1** influences both the α3 and L3 regions (Figures S22–24). Interestingly, however, the results imply that **1** binds to SPM‐1 in the presence of ML302F, which is known to bind to the active site zinc ions.^17^ Together with the observation that **1** binds to *apo*‐Y58C* as evidenced by line broadening (Figure 2 C), the results imply that **1** binds to SPM‐1 in an unprecedented manner that does not involve coordination to the zinc ions.

We then tested the utility of PrOF NMR for monitoring the binding of weak SPM‐1 inhibitors, as exemplified by avibactam, which inhibits class A, C, and some D β‐lactamases,^3^, 4c but has low affinity for most MBLs.4b A clear chemical‐shift change was observed with avibactam and Y58C* but not F151C*, thus indicating that avibactam binding induces changes in the L3 region but not the α3 region (Figures S25,26). With Y58C*, a shift back to the original protein peak was observed after 12 h, likely as a result of slow hydrolysis of avibactam catalyzed by SPM‐1.4b Addition of fresh avibactam to the reacted solution shifted the peak towards that arising originally from avibactam with Y58C*.

We then investigated the addition of β‐lactam substrates [a carbapenem (meropenem), a penicillin (piperacillin), and mechanism‐based inhibitors of class A β‐lactamases (tazobactam and clavulanic acid)] to the SPM‐1* variants. Their addition to SPM‐1* caused line broadening and chemical‐shift changes for Y58C* but not (within detection limits) for F151C* (Figure 3).8b Meropenem treatment (400 μm) of Y58C* (40 μm) led to a 0.2 ppm ^19^F shift (from −83.15 ppm to −82.95 ppm), thus implying fast exchange (Figure 3 A,E). Time‐course analysis revealed spectra that are stable for 12 h (Figure 3 C), thus suggesting that the new peak likely reflects an enzyme–product complex (Figures S27–31). With piperacillin (400 μm), a shift of 0.4 ppm was also observed (Figure 3 B,F). However, in contrast to meropenem, time‐course analysis revealed additional line broadening and a further chemical shift of 0.18 ppm relative to the product complex peak from −82.75 ppm to −82.57 ppm (Figure 3 D and Figure S32), thus indicating production of a new SPM‐1 binding species.

**Figure 3:**
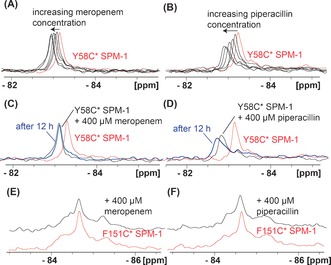
Interactions of (hydrolyzed) β‐lactams with SPM‐1* variants as analyzed by by PrOF NMR. Titration of A) meropenem and B) piperacillin with Y58C* SPM‐1 reveals interactions with the L3 region. Time‐course analyses (after 12 h) of C) meropenem and D) piperacillin with Y58C* SPM‐1 are consistent with a stable protein–product peak with an additional shift in the case of piperacillin, which indicates the formation of a new species. Titration of E) meropenem and F) piperacillin into a solution of F151C* SPM‐1 shows no substantial changes. Assay mixtures: 40 μm SPM‐1* and increasing ligand concentrations (up to 400 μm) in 50 mm Tris, pH 7.5, 9:1 H_2_O/D_2_O. For Δ*δ*_max_<0.1 ppm, observations are denoted as “no substantial changes”.

Previous work has revealed that the product of piperacillin hydrolysis can bind to penicillin‐binding proteins, with the “epimerized” (5*S*)‐product binding in preference to the initially formed (5*R*)‐penicilloic acid (PA).^20^ We thus used ^1^H NMR to evaluate the time‐dependent SPM‐1‐catalyzed hydrolysis of piperacillin (Figure S33). The results reveal that SPM‐1 catalyzes piperacillin hydrolysis to give (5*R*)‐PA, which epimerizes relatively slowly to give (5*S*)‐PA, likely through a non‐enzyme‐catalyzed pathway. To investigate binding of (5*S*)‐PA and (5*R*)‐PA to SPM‐1, the *Bacillus cereus* BcII MBL^14^ was used to produce PA from piperacillin, which was then purified. Addition of the resultant (5*S*)/(5*R*)‐PA mixture to Y58C* led to a peak at −82.57 ppm, as observed after 12 h in the piperacillin time course (Figure 4). ^1^H and water LOGSY analyses revealed binding of both (5*S*)‐PA and (5*R*)‐PA to SPM‐1 (Figure S34).

**Figure 4:**
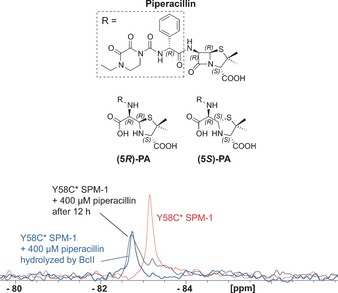
^19^F‐NMR spectra of Y58C* SPM‐1 interacting with hydrolyzed piperacillin. The structures of piperacillin and its hydrolyzed products [(5*R*)‐PA and (5*S*)‐PA] are shown. Assay mixtures: 40 μm Y58C* SPM‐1 and 400 μm added ligand in 50 mm Tris, pH 7.5, 9:1 H_2_O/D_2_O.

We then used PrOF NMR to investigate interactions of SPM‐1 with the class A SBL inhibitors clavulanic acid and tazobactam, which are SPM‐1 substrates.8a Line broadening and a shift from −83.15 to −83.02 and −82.98 ppm were observed in the ^19^F Y58C* spectra for tazobactam and clavulanic acid, respectively; no further substantial changes were evident after 12 h. No such effects were observed for F151C* (Figures S35–39). The propensity of clavulanic acid and tazobactam to undergo complex fragmentations^21^ (as observed with SBLs) precluded identification of the species that give rise to these shifts. In the case of clavulanic acid, ^1^H NMR studies (Figure S40) indicated the formation of multiple products, only some of which likely bind to SPM‐1.

The overall results reveal the importance of the dynamic α3 and L3 regions in ligand binding by SPM‐1. They also illustrate how PrOF NMR can reveal previously unidentified binding modes, as observed with isoquinoline (**1**). All of the inhibitors tested, including the Zn^II^ chelator 1,10‐*o*‐phenanthroline, bring about substantial changes in both the α3 and L3 regions, thus emphasizing the importance of both, particularly the α3 region, in inhibitor development. In contrast, the β‐lactam substrates (piperacillin, meropenem, tazobactam, and clavulanate) give rise to hydrolyzed products which bring about changes in the L3 region. Although it is possible that substrate binding involves both the α3 and L3 regions, the latter is more important in product binding, and hence likely in product release too. This is consistent with the proposal indicating that SPM‐1 is mechanistically closer to the B1 rather than B2 MBLs,8b based on work showing that deletion of the SPM‐1 α3–α4 region does not substantially affect β‐lactam hydrolysis,8a and crystallographic studies on the B1 MBL NDM‐1 implying that binding of hydrolyzed meropenem involves the L3 region (Figure S31).^22^ With the penicillin substrates, we observed binding of both (5*R*)‐PA and (5*S*)‐PA, thus illustrating the utility of PrOF NMR for studying the binding of equilibrating mixtures of stereoisomers. The observation of penicilloic acid and hydrolyzed meropenem binding to SPM‐1 is of potential clinical relevance. Previous studies have shown that penicilloic acids are competitive inhibitors of serine β‐lactamases^23^ and MBLs.^24^ Although the levels of inhibition by penicilloic acids are much less than those for the intact β‐lactams, given the high concentration of β‐lactams used clinically, it is possible that β‐lactamase inhibition by PAs is relevant. The results are of interest for identifying novel inhibitor scaffolds for SPM‐1 and other MBLs, including the design of non‐β‐lactam inhibitors that are not susceptible to β‐lactamase hydrolysis, and/or β‐lactams or β‐lactam analogues that give hydrolyzed products that inhibit MBLs. Whist ^13^C/^15^N labeling is often powerful for studying ligand binding, it is relatively expensive and time consuming. In contrast, our results clearly illustrate the utility of PrOF NMR for studying protein–ligand interactions in solution, detecting metal chelation, and revealing subtle differences in binding modes.

## Conflict of interest

The authors declare no conflict of interest.

## Supporting information

As a service to our authors and readers, this journal provides supporting information supplied by the authors. Such materials are peer reviewed and may be re‐organized for online delivery, but are not copy‐edited or typeset. Technical support issues arising from supporting information (other than missing files) should be addressed to the authors.

Supplementary
