# Supplementary material for: 19F‐NMR Reveals the Role of Mobile Loops in Product and Inhibitor Binding by the São Paulo Metallo‐β‐Lactamase
Source: Angew Chem Int Ed Engl. 2017 Mar 2;56(14):3862–6. doi: 10.1002/anie.201612185 (PMC5396265; doi:10.1002/anie.201612185)
Supplement: Supplementary file 1 — Supplementary [file ANIE-56-3862-s001.pdf]

Angewandte Chemie
Eine Zeitschrift der Gesellschaft Deutscher Chemiker

Supporting Information

^{19}F-NMR Reveals the Role of Mobile Loops in Product and Inhibitor Binding by the São Paulo Metallo-β-Lactamase

Martine I. Abboud, Philip Hinchliffe, Jürgen Brem, Robert Macsics, Inga Pfeffer, Anne Makena, Klaus-Daniel Umland, Anna M. Rydzik, Guo-Bo Li, James Spencer, Timothy D. W. Claridge, and Christopher J. Schofield**

anie_201612185_sm_miscellaneous_information.pdf

Table of Contents

MATERIALS AND METHODS.. 5

Reagents.. 5

Mutagenesis... 5

Expression Trials and Protein Production 5

Fluorine Labeling 6

***Apo*-SPM-1 Production 6**

Protein Electrospray Ionization Mass Spectrometry 6

In-Solution Trypsin Digestion 7

MALDI-ToF-MS and MS/MS Studies 7

Circular Dichroism (CD) Experiments 7

Crystallization Conditions 8

Steady-State Kinetics... 8

Synthesis 9

Production of Hydrolyzed β-Lactams 9

NMR Experiments.. 9

^{19}F-NMR Experiments 9

^{1}H CPMG NMR Experiments 9

wLOGSY NMR Experiments 10

SUPPLEMENTARY FIGURES.. 11

Fig. S1. Views from MBL crystal structures highlighting potentially mobile regions. 11

Fig. S2. Outline reaction of BTFA labeling and an active site view from an SPM-1 crystal structure. 12

Fig. S3. View from an SPM-1 crystal structure showing the differences in the location of residues Y152 and F151 in the α3 region.. 13

Fig. S4. Y58C SPM-1 protein purification 14

Fig. S5. F151C SPM-1 protein purification... 15

Fig. S6. MS spectra of unmodified Y58C SPM-1 (upper spectrum) and BTFA-labeled Y58C SPM-1 (lower spectrum). 16

Fig. S7. MS spectra of unmodified F151C SPM-1 (left) and BTFA-labeled F151C SPM-1 (right). 17

Fig. S8. MALDI-ToF MS spectra of BTFA-labeled (blue) and unlabeled (red) Y58C SPM-1 after digestion with porcine trypsin. 18

Fig. S9. MALDI-ToF MS spectra of BTFA-labeled (blue) and unlabeled (red) Y58C SPM-1 after digestion with porcine trypsin (close-up). 19

Fig. S10. MALDI-ToF MS spectrum of the BTFA-labeled F151C SPM-1 variant after digestion with porcine trypsin. 20

Fig. S11. MALDI-ToF MS spectra of BTFA-labeled F151C SPM-1 variant after digestion with porcine trypsin (close-up). 21

Fig. S12. CD spectra of SPM-1 variants. 22

Fig. S13. View from Y58C SPM-1 variant crystal structure. 23

Fig. S14. Overlay of wildtype SPM-1 and Y58C SPM-1 variant crystal structures. 24

Fig. S15. Meropenem turnover by wt SPM-1, Y58C* SPM-1, and F151C* SPM-1. 25

Fig. S16. PrOF NMR spectra of *apo*- and di-Zn(II)-SPM-1* variants. 25

Fig. S17. Solvent exposure studies of SPM-1* variants by PrOF NMR. 26

Fig. S18. DMSO titration studies with SPM-1* variants by PrOF NMR. 26

Fig. S19. ^{19}F-NMR time-course spectra of SPM-1* variants treated with 1,10-*o*-phenanthroline. 27

Fig. S20. ^{1}H CPMG NMR spectra of 1,10-o-phenanthroline treated with SPM-1* variants…………… 27

Fig. S21. PrOF NMR analyses of ML302 and ML302F and SPM-1* variants. 28

Fig. S22. ^{19}F-NMR spectra of L-captopril and SPM-1* solutions. 29

Fig. S23. ^{19}F-NMR time-course spectra of SPM-1* variants with the isoquinoline derivative (1). 30

Fig. S24. ^{1}H CPMG NMR spectra of the isoquinoline derivative (1) with SPM-1* variants. 30

Fig. S25. ^{19}F-NMR spectra of avibactam and Y58C* SPM-1. 31

Fig. S26. ^{19}F-NMR spectra of avibactam and F151C* SPM-1. 31

Fig. S27. ^{19}F-NMR time-course spectra of F151C* SPM-1 with meropenem. 32

Fig. S28. ^{1}H CPMG NMR spectra of meropenem with SPM-1 variants. 32

Fig. S29. Monitoring the binding of the products of meropenem hydrolysis to SPM-1 by ^{1}H and wLOGSY NMR. 33

Fig. S30. ^{19}F-NMR of Y58C* SPM-1 interaction with hydrolyzed meropenem. 33

Fig. S31. View from NDM-1 crystal structure in complex with hydrolyzed meropenem. 34

Fig. S32. ^{19}F-NMR time-course spectra of piperacillin with F151C* SPM-1. 34

Fig. S33. Monitoring the reaction of piperacillin with SPM-1 after 12 h. 35

Fig. S34. Monitoring the binding of hydrolyzed piperacillin to SPM-1 by ^{1}H and wLOGSY NMR. 36

Fig. S35. ^{19}F-NMR spectra of the titration of tazobactam into Y58C* SPM-1 (A) and F151C* SPM-1 (B) solution. 37

Fig. S36. ^{19}F-NMR time-course spectra of SPM-1* variants with tazobactam. 38

Fig. S37. ^{1}H CPMG NMR spectra of tazobactam with SPM-1* variants. 38

Fig. S38. ^{19}F-NMR spectra of the titration of clavulanic acid into Y58C* SPM-1 (A) or F151C* SPM-1 (B) solutions. 39

Fig. S39. ^{19}F-NMR time-course spectra of SPM-1* variants with clavulanic acid. 40

Fig. S40. ^{1}H NMR spectra of the reaction of clavulanic acid with SPM-1. 41

SUPPLEMENTARY TABLES 42

Table S1. Data collection and refinement statistics. 42

Table S2. Table summarizing the NMR observations with SPM-1. 43

Table S3. Table summarizing the K_D values of the ligands to SPM-1* as observed by PrOF NMR. 43

REFERENCES 48

Materials and Methods

Reagents

Tazobactam, meropenem, and sodium piperacillin were from Molekula. Avibactam was a gift from AstraZeneca. Unless otherwise stated, other reagents were from Sigma-Aldrich.

Mutagenesis

The Y58C SPM-1 and F151C SPM-1 variants were generated using the wildtype (wt) pOPINF SPM-1 plasmid.[1] The desired point mutations were achieved employing the QuikChange site directed mutagenesis kit (Stratagene) using the following primers:

SPM-1 variant	Primers sequences
**Y58C SPM-1**	Forward: 5'-cagcacgttggaactgcagaagtcacgatcggt-3'<br>Reverse: 5'-accgatcgtgacttctgcagttccaacgtgctg-3'
**F151C SPM-1**	Forward: 5'-aggtcttcattcttgtaacattcggccgctttaatgcg-3<br>Reverse: 5'-cgcattaaagcggccgaatgttacaagaatgaagacct-3'

Expression Trials and Protein Production

Expression trials were performed at different temperatures (18, 30, 37 ºC) using various isopropyl β-D-1-thiogalactopyranoside (IPTG) concentrations (0, 0.1, 0.2, 0.5, 0.7 and 1mM) and different induction times (4, 6, 8 and 20 h). Cells were lysed using the BugBuster kit; preferred conditions for expression were assessed by SDS-PAGE analyses. Recombinant di-Zn(II)-SPM-1 proteins were produced in *E. coli* BL21(DE3) pLysS cells using 2TY medium supplemented with 50 μg/mL ampicillin and 34 μg/mL chloramphenicol. Cells were grown at 37 ºC until they reached OD_{600} = 0.6-0.8; the temperature was then reduced to 30 ºC for 6 h. Cells were harvested by centrifugation (10 min, 10g), then resuspended in 50 mL lysis buffer supplemented with DNaseI, lysozyme and EDTA-free protease-inhibitors. The cells were then further lysed by sonication (2 x 7 min); cell debris was then removed by centrifugation (20 g, 30 min). The cell lysates were then loaded onto a 5 mL HisTrap HP column (GE Healthcare Life Sciences, Little Chalfont, UK), with 50 mM Tris pH 7.5, 500 mM NaCl, containing 20 mM imidazole, then eluted with an imidazole gradient (up to 500 mM imidazole). Fractions containing di-Zn(II)-SPM-1 were further purified using a Superdex S200 column (300 mL) equilibrated with 20 mM Tris, pH 7.5, 200 mM NaCl. Eluted SPM-1 fractions were incubated overnight at 4 °C with 3C protease (1:100 w/w) and then purified using a 5 mL HisTrap HP column to give the untagged protein. The

purity of the resulting fractions was assessed as > 90% (by SDS-PAGE). Fractions containing purified protein were concentrated by centrifugal ultra-filtration (10 kDa cut-off membrane). The concentrations of the purified proteins were determined using a ND-1000 NanoDrop spectrophotometer, as previously reported.

Fluorine Labeling

SPM-1 variants (Y58C and F151C) were incubated on ice with tris-(2-carboxyethyl)phosphine (TCEP, final concentration 2 mM) in 50 mM Tris buffer, pH 7.5, for 30 min. The samples were buffer exchanged into phosphate buffer (50 mM, pH 7.0, 200 mM NaCl) using a PD-10 desalting column (GE Healthcare). A concentrated stock of 100 mM 3-bromo-1,1,1-trifluoroacetone (BTFA) reagent in phosphate buffer was prepared freshly prior to the reaction. SPM-1 variant samples were treated with 35-fold equivalent of 3-bromo-1,1,1-trifluoroacetone for 10 min at room temperature, prior to buffer exchange into Tris buffer (50 mM, pH 7.5, 200 mM NaCl) using a PD-10 desalting column (GE Healthcare).[2]

Apo-SPM-1 Production

Apo-SPM-1 proteins were produced by EDTA treatment of the di-Zn(II)-SPM-1. The di-Zn(II)-SPM-1 variants were incubated with 1,000-equivalents of EDTA at 4 ºC overnight. The treated proteins were purified using a Superdex S200 column (300 mL) equilibrated with 20 mM Tris pH 7.5, 200 mM NaCl. Fractions containing purified protein were concentrated by centrifugal ultrafiltration (10 kDa cutoffs). The purity of the resulting fractions was ascertained to be > 90% by SDS-PAGE. Concentrations of the purified proteins were determined using a ND-1000 NanoDrop spectrophotometer. Removal of the metal was confirmed by non-denaturing mass spectrometry and activity analyses.

Protein Electrospray Ionization Mass Spectrometry

Low resolution positive ion electrospray mass spectra were recorded using an LCT Premier XE ionization (Micromass®) mass spectrometer interfaced with a Acquity™ Ultra Performance liquid chromatography (UPLC) system using a Acquity™ UPLCR BEH300 C18 column at 50 ºC (Waters Corporation). The protein (1 ng.mL^{-1}, 0.5 μL) was injected and eluted at 0.3 mL.min^{-1} using a gradient system from Solvent A (95 % water, 0.1 % (v/v) formic acid) to Solvent B (95 % acetonitrile, 0.1 % (v/v) formic acid). The eluent was injected directly into the mass spectrometer. The following MS parameters were used: capillary voltage, 3,000 V; sample cone voltage, 35 V; desolvation temperature, 250 ºC; source temperature, 80 ºC; cone gas flow, 100 L.h^{-1} and desolvation gas flow (N_2), 400 L.h^{-1}.

Sodium formate was used as a calibrant. Spectra were processed using MassLynx™ v4.0 and v4.1 (Waters Corporation) with the Maximum Entropy method (MaxEnt1). Calculated masses were obtained using the ExPasy ProtParam tool (http://web.expasy.org/protparam/).

In-Solution Trypsin Digestion

All reagents were prepared in Tris buffer (100 mM, pH 7.8). Samples (< 500 µg) were dried in a Vacufuge® vacuum concentrator (*Eppendorf*) connected to an external diaphragm pump and then resuspended in 6 M urea (100 µL). Disulfides were reduced at room temperature (30 min) by addition of dithiothreitol (DTT, 5 µL, 200 mM), and subsequently alkylated at room temperature (30 min) with iodoacetamide (30 µL, 200 mM).[2-3] Unreacted alkylating reagent was quenched by addition of aqueous DTT (30 µL, 30 min). The sample was then diluted with Tris buffer (775 µL), mixed with Sequencing Grade Modified Porcine Trypsin (*Promega*) at trypsin:protein sample ratio of 1:50, and digested at 37°C for 12 h. Digestion was stopped by adjusting the sample to pH 3-4 by addition of concentrated acetic acid. Digested samples were subsequently purified and desalted by solid-phase extraction using Sep-Pak C18 Plus Light Cartridges (*Waters*, 130 mg sorbent per cartridge, 55-105 µM particle size) following the manufacturer's protocol. The samples were then dried using a Vacufuge® concentrator and subsequently re-dissolved in (typically) 50% CH_3CN/0.1% CF_3COOH (15-30 µL) for analysis by MALDI-ToF-MS.

MALDI-ToF-MS and MS/MS Studies

Matrix-assisted laser desorption/ionization time of flight mass spectrometry (MALDI-ToF-MS) and MS/MS analyses were performed using a *Bruker Daltonics* Ultraflex™ MALDI-ToF/ToF machine, using flexControl™ 3.0 software. Spectra were recorded in the positive ion reflectron mode, typically with 32-38 % laser energy. Calibration was performed on each day prior to the measurements using Peptide Calibration Standard II (*Bruker Daltonics*). Data were processed using *Bruker Daltonics* flexAnalysis™ 3.0 software and assigned manually. For MALDI measurements, the sample (1 µL) was mixed with 2,5-dihydroxybenzoic acid (4 µL, DHB, 20 mg/mL in 50 % CH_3CN/0.1 % CF_3COOH) matrix and this sample-matrix mixture spotted (2 µL) onto a 24 x 16 MTP AnchorChip™ 384 ToF MALDI target and allowed to air-dry before analysis.

Circular Dichroism (CD) Experiments

CD measurements were carried out using a Chirascan CD spectrometer (Applied Photophysics) equipped with a Peltier temperature-controlled cell holder. Experiments were

performed at 23 °C in a 0.1 cm path length cuvette using 0.2 mg/mL protein in 10 mM sodium phosphate buffer (pH 8.0) supplemented with 50 mM $ZnSO_4$. Data were recorded from 260 to 185 nm at 0.5 nm intervals; each data point was averaged for 1 s. Spectra were baseline corrected and smoothed using the Savitzky–Golay filter. Data recorded in the 190–240 nm range were analyzed using DichroWeb; the CDSSTR deconvolution method was used to estimate secondary structural content using reference set 4. To minimize the effects of differences in protein concentration, the data were normalized at 207 nm.[4]

Crystallization Conditions

SPM-1 Y58C (20 mg/mL) was crystallized using sitting-drop vapor diffusion in 24-well Cryschem plates (Hampton Research). Protein (2 µL) was mixed with crystallization reagent (0.1 M mixture of HEPES and MOPS, pH 7.5, 0.03 M NaF, 0.03 M NaI, 0.03 M NaB, 12% PEG3350, 12% PEG1000, 12% 2-methyl-2,4-pentanediol) and equilibrated against 500 µL. Crystals grew to maximum size in 3 days and were looped directly from the drop and flash-frozen in liquid nitrogen.

Diffraction data were collected at 100 K on beamline I24 (Diamond Light Source, Didcot, UK). The dataset was indexed and integrated using XDS[5] and scaled using Aimless in CCP4.[6] Data were cut at 1.75 Å as completeness dropped significantly at higher resolutions. Phases were determined using molecular replacement in Phaser[7] with 4BP0[2] as the search model (with Y58 mutated to a cysteine). The structure was refined and modelled using Phenix[8] and Coot,[9] respectively. After several rounds of refinement residue 58 was modelled as a cysteine into clearly defined electron density, before undergoing a final round of refinement and model building. Structure validation was assisted by Molprobity[10] and Phenix. Data collection and refinement statistics are given in Table S1.

Steady-State Kinetics

Hydrolysis of meropenem was monitored at 25 °C in 50 mM HEPES buffer (pH 7.2) supplemented with 1 µg/mL BSA, 1 mM $ZnSO_4$ and 0.01% Triton X-100. Analyses were carried out in triplicate ($n \geq 3$); the absorbance values were read using a BMG Labtech Pherastar FS plate reader at 300 nm. Extinction coefficients were determined by plotting the absorbance units against decreasing concentrations of the substrate. Kinetic constants (K_M and k_{cat}) were obtained by determining the initial rate of the reaction at different substrate concentrations. The concentration-dependence of the initial rate was fitted and analyzed using GraphPad Prism 5.01 software to generate Michaelis–Menten curves.[11]

Synthesis

ML302 and ML302F were synthesized as reported by Brem *et al.*[12] The isoquinoline derivative **(1)** was synthesized as reported by van Berkel *et al.*[11]

Production of Hydrolyzed β-Lactams

β-Lactamase mediated hydrolysis was used to produce hydrolyzed meropenem and piperacillin. Incubation of 5 equivalents of piperacillin and meropenem with *Bacillus cereus* m569/H/9 (BcII) produced (5*R*)-penicilloic acid (PA) [and some (5*S*)-PA] and hydrolyzed meropenem, respectively. BcII was removed from the reaction mixture using PD-10 columns. Subsequent epimerisation of (5*R*)- to (5*S*)-PA was performed under neutral conditions, non-enzymatically, at 4°C, for an overnight. The hydrolyzed products were obtained *via* spin filtration using 10 kDa Centricon concentrators. The reaction and the purity of hydrolyzed β-lactams were assessed by NMR analyses. BcII was produced and purified as described by van Berkel *et al.*[11]

NMR Experiments

Spectra were recorded using a Bruker AVIII 600 with BB-^{19}F/^{1}H Prodigy N_2 cryoprobe operating at 298 K using 5 mm diameter NMR tubes (Norell).

^{19}F-NMR Experiments

^{19}F NMR experiments were typically obtained using 256 scans and a recovery delay of 2 s. Data were processed with 5 Hz Lorentzian line broadening using TopSpin 3.1 software (Bruker) and were referenced to the internal 1,1,1-trifluoroacetic acid standard (TFA, 10 µM, at -74.45 ppm). Samples contained the SPM-1* variant (40 µM, unless otherwise stated) and added ligand in Tris buffer (50 mM, pH 7.5) supplemented with 10% D_2O.

^{1}H CPMG NMR Experiments

Typical experimental parameters for Carr-Purcell-Meiboom-Gill (CPMG) NMR spectroscopy were as follows: total echo time, 40 ms; relaxation delay, 2 s; number of transients, 64. The PROJECT-CPMG sequence (90°x−[τ−180°y−τ−90°y−τ−180°y−τ]*n*−acq) was applied.[13] Water suppression was achieved by pre-saturation. Data were processed with 0.3 Hz Lorentzian line broadening using TopSpin 3.1 software (Bruker). Unless otherwise stated, assay mixtures contained 40 µM protein and 400 µM of ligand buffered with 50 mM Tris-D_{11} (pH 7.5) and 0.02 % NaN_3 in 90 % H_2O and 10 % D_2O.

wLOGSY NMR Experiments

For water-Ligand Observed Gradient SpectroscopY (wLOGSY) analyses,[14] typical experimental parameters were: mixing time, 1 s; relaxation delay, 2 s; number of transients, 256. Solvent excitation was achieved using a 16 ms 180 degrees selective rectangular shape pulse with 1000 points (Squa100.1000) set at the H_2O frequency. Water suppression was achieved by a 2 ms Sinc pulse (Sinc1.1000) at the H_2O frequency. Data were processed with 0.3 Hz Lorentzian line broadening using TopSpin 3.1 software (Bruker). Unless otherwise stated, assay mixtures contained 40 µM protein and 400 µM of ligand buffered with 50 mM Tris-D_{11} (pH 7.5) and 0.02 % NaN_3 in 90 % H_2O and 10 % D_2O.

Supplementary Figures

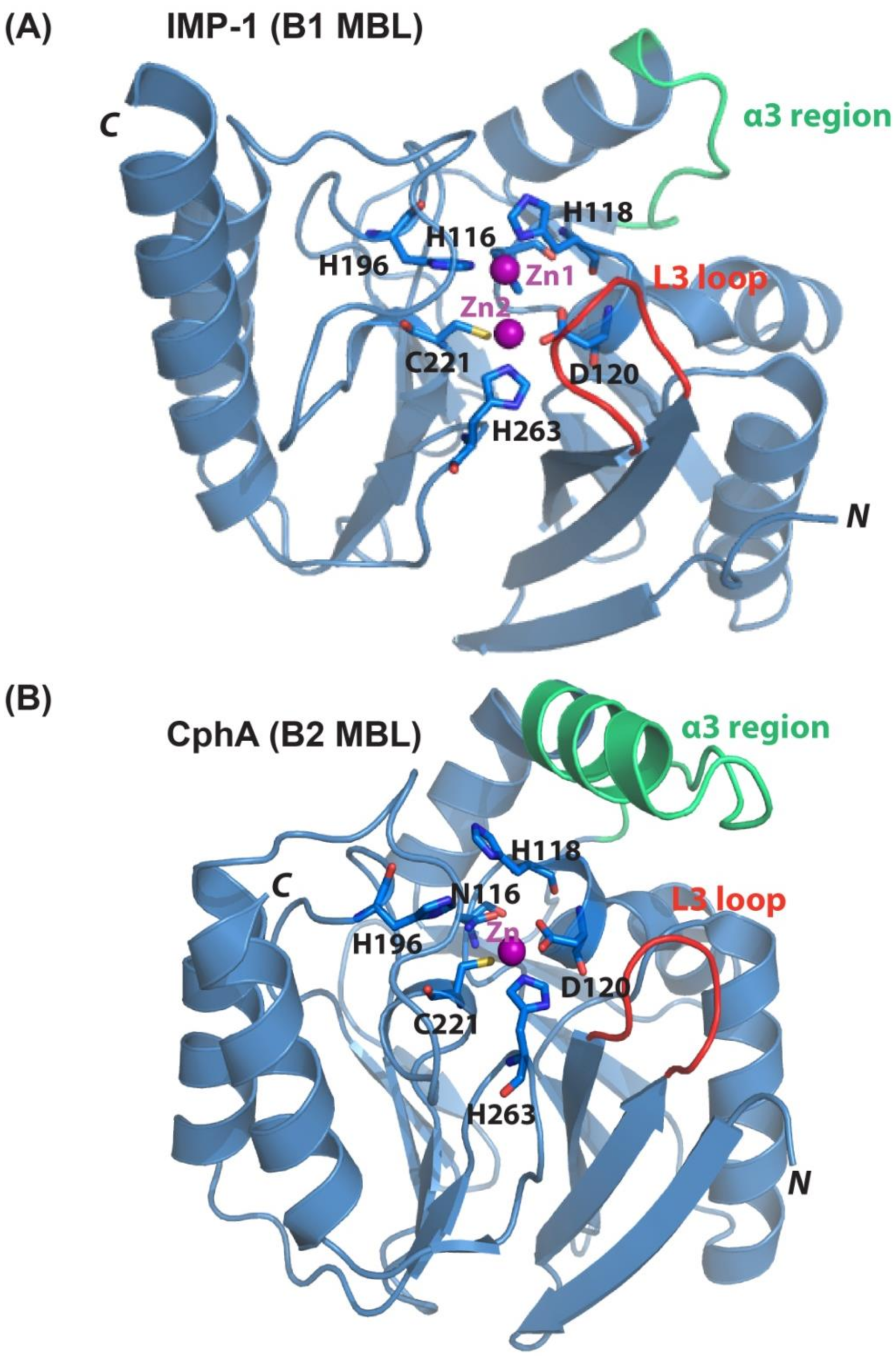

Fig. S1. Views from MBL crystal structures highlighting potentially mobile regions. Views from structures of **(A)** IMP-1 (a di-Zn(II) binding B1 MBL, PDB ID: 1JJT)[15] and **(B)** CphA (a mono-Zn(II) binding B2 MBL, PDB ID: 1X8I)[16] highlighting the different mobile regions [L3 loop (red), and α3 region (green)] that characterize the MBL subfamilies. A longer L3 loop (red) is characteristic of the di-Zn(II) B1 MBLs. The mono-Zn(II) binding B2 MBLs are (normally) characterized by an elongated α3 region (green) and a shorter L3 loop (red).[17]

(A)

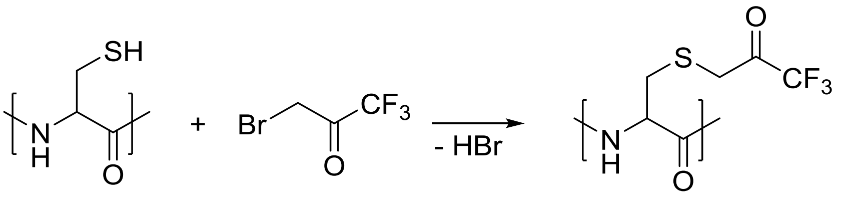

(B)

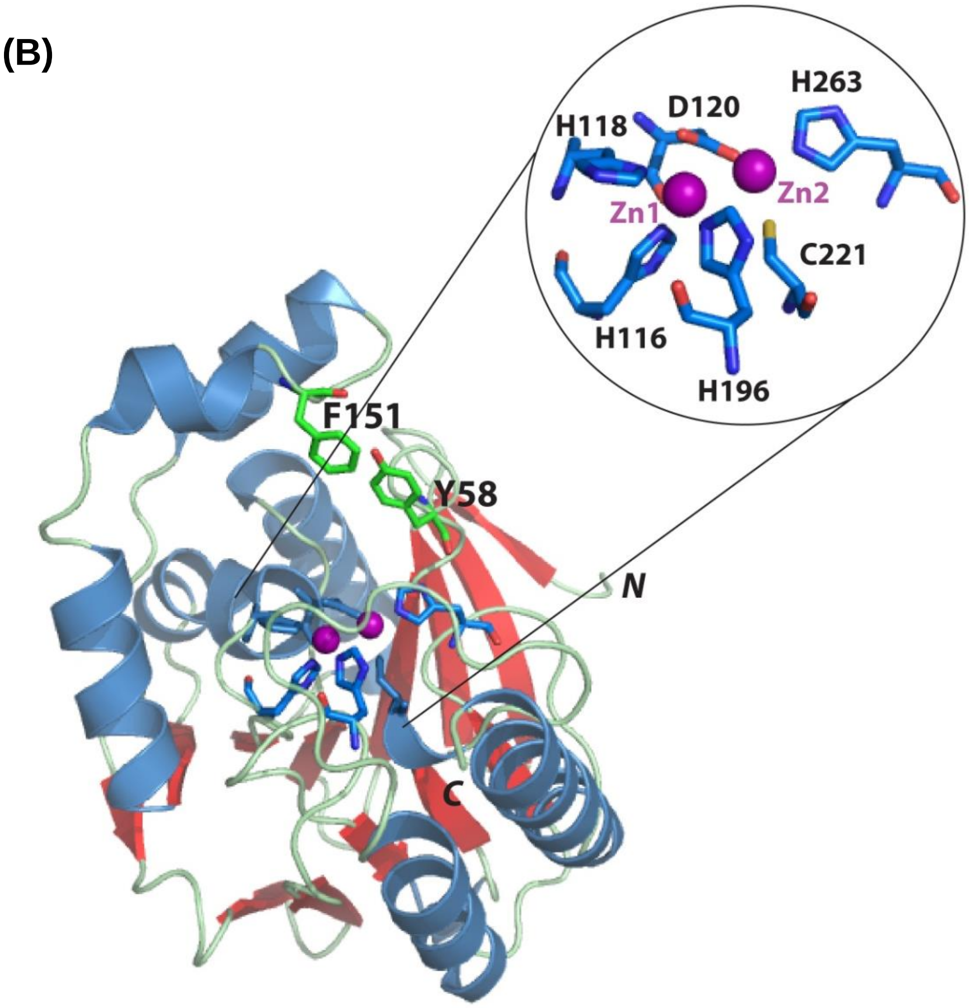

Fig. S2. Outline reaction of BTFA labeling and an active site view from an SPM-1 crystal structure. PDB ID: 4BP0.[3] The sites of labeling, F151 and Y58, are highlighted in green. Selected active site residues are shown as sticks in the highlighted circle. Zn1 is coordinated by 3 His residues, H116, H118, and H196, and Zn2 is coordinated by D120, H263, and C221. The figure was created using PyMOL.

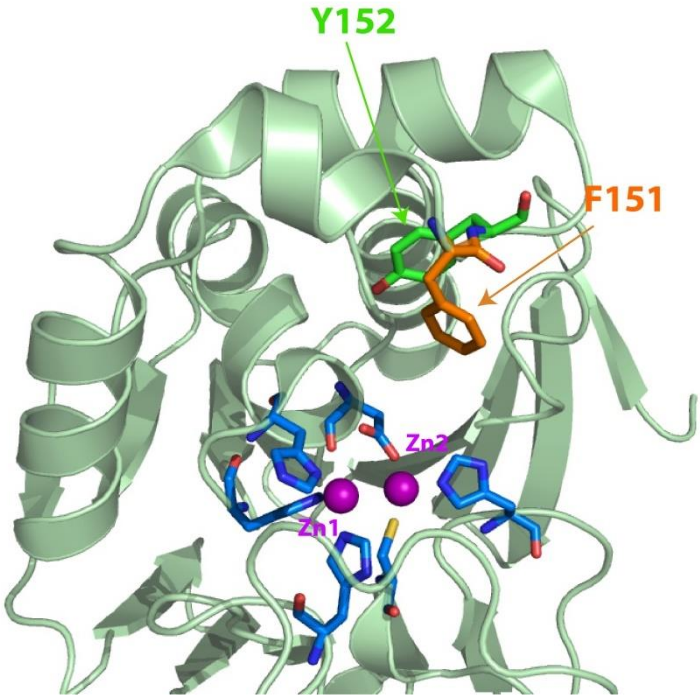

Fig. S3. View from an SPM-1 crystal structure showing locations of residues Y152 and F151 in the α3 region. PDB ID: 4BP0.[18] The figure was created using PyMOL.

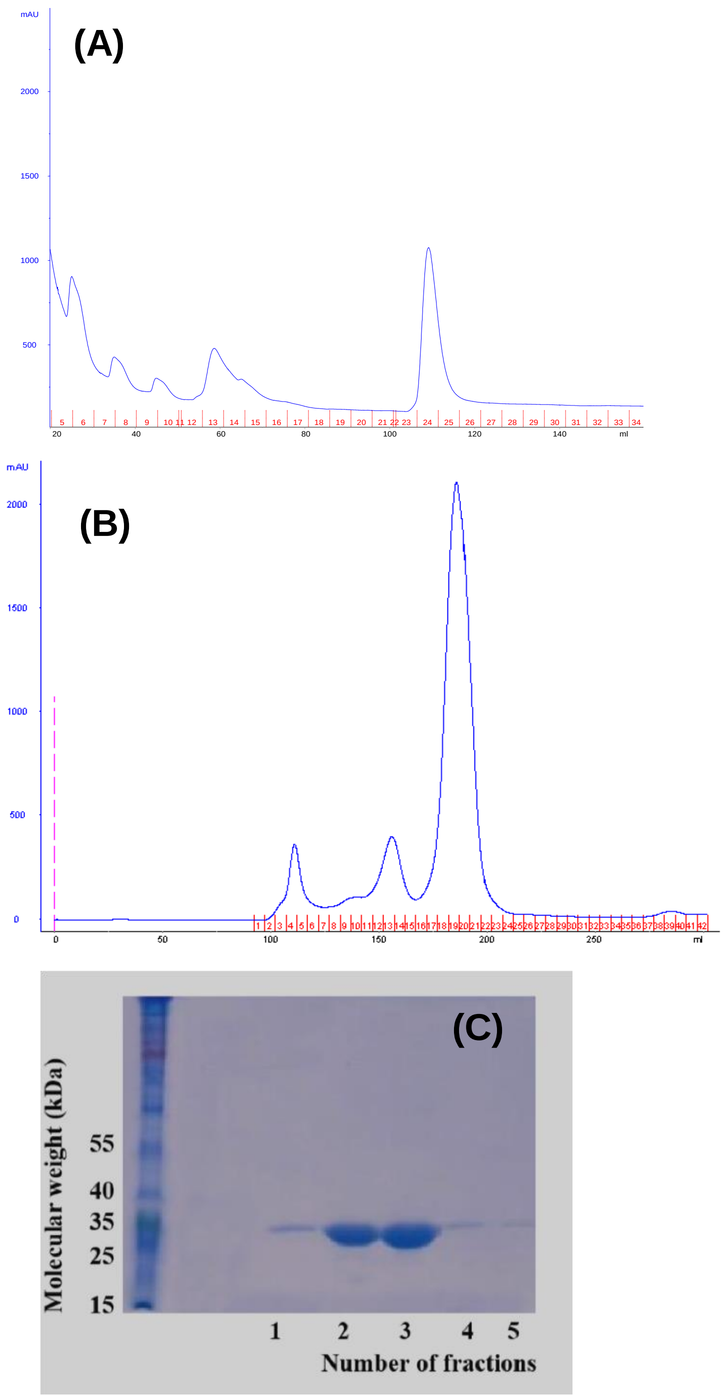

Fig. S4. Y58C SPM-1 protein purification by: **(A)** affinity chromatography (5 mL HisTrap HP column), **(B)** gel filtration (S75 300 mL preparative column), and **(C)** SDS-PAGE after His-tag cleavage. FPLC traces show UV absorbance in mAU versus volume eluted in mL. SDS-PAGE Lane 1 – molecular weight markers (PageRuler Prestained Protein Ladder 10-170 kDa, Thermo Scientific).

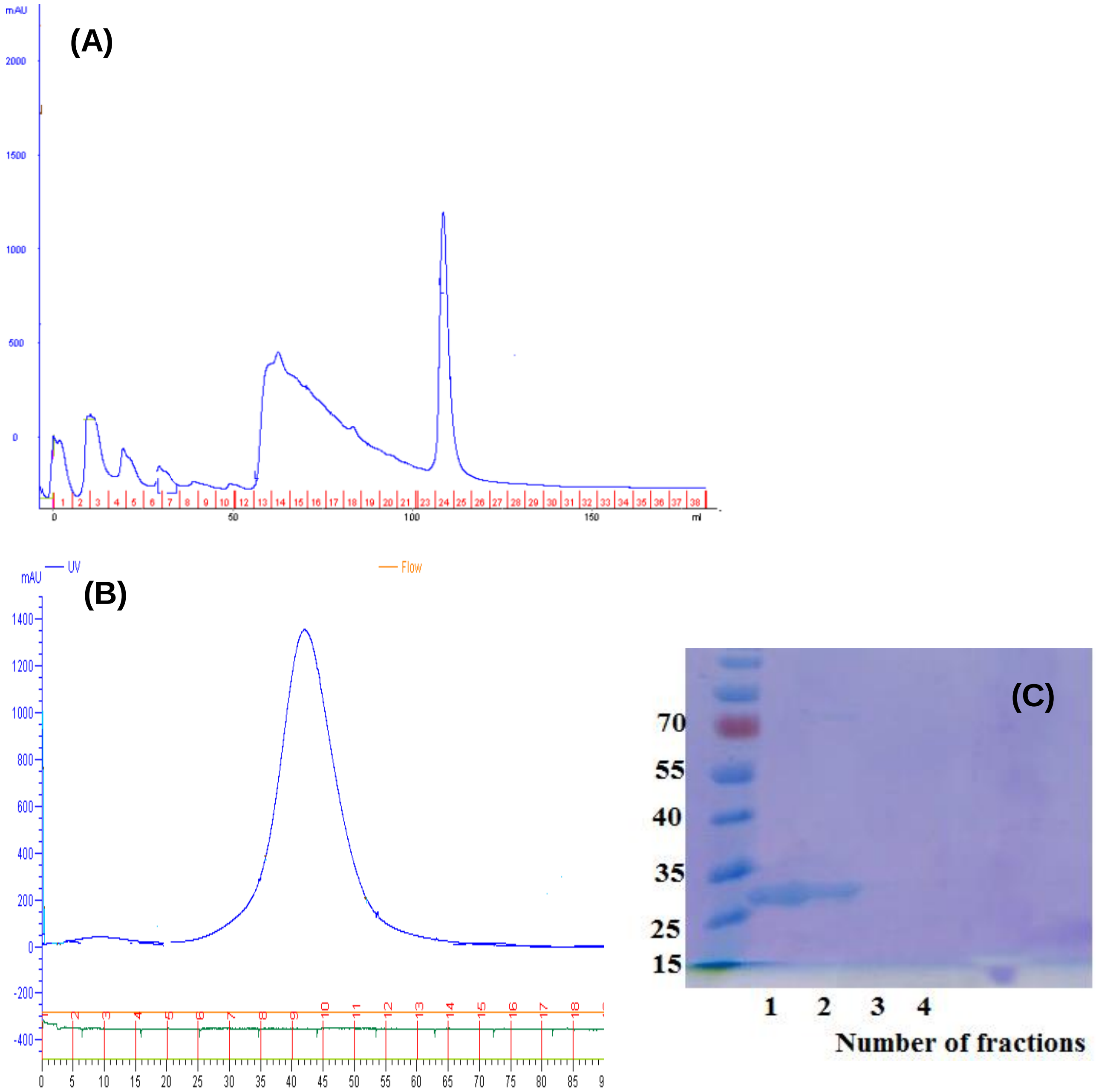

Fig. S5. F151C SPM-1 protein purification by: **(A)** affinity chromatography (5 mL HisTrap HP column), **(B)** gel filtration (S75 150 mL preparative column), and **(C)** SDS-PAGE after His-tag cleavage. FPLC traces show UV absorbance in mAU versus volume eluted in mL. SDS-PAGE Lane 1 – molecular weight markers (PageRuler Prestained Protein Ladder 10-170 kDa, Thermo Scientific).

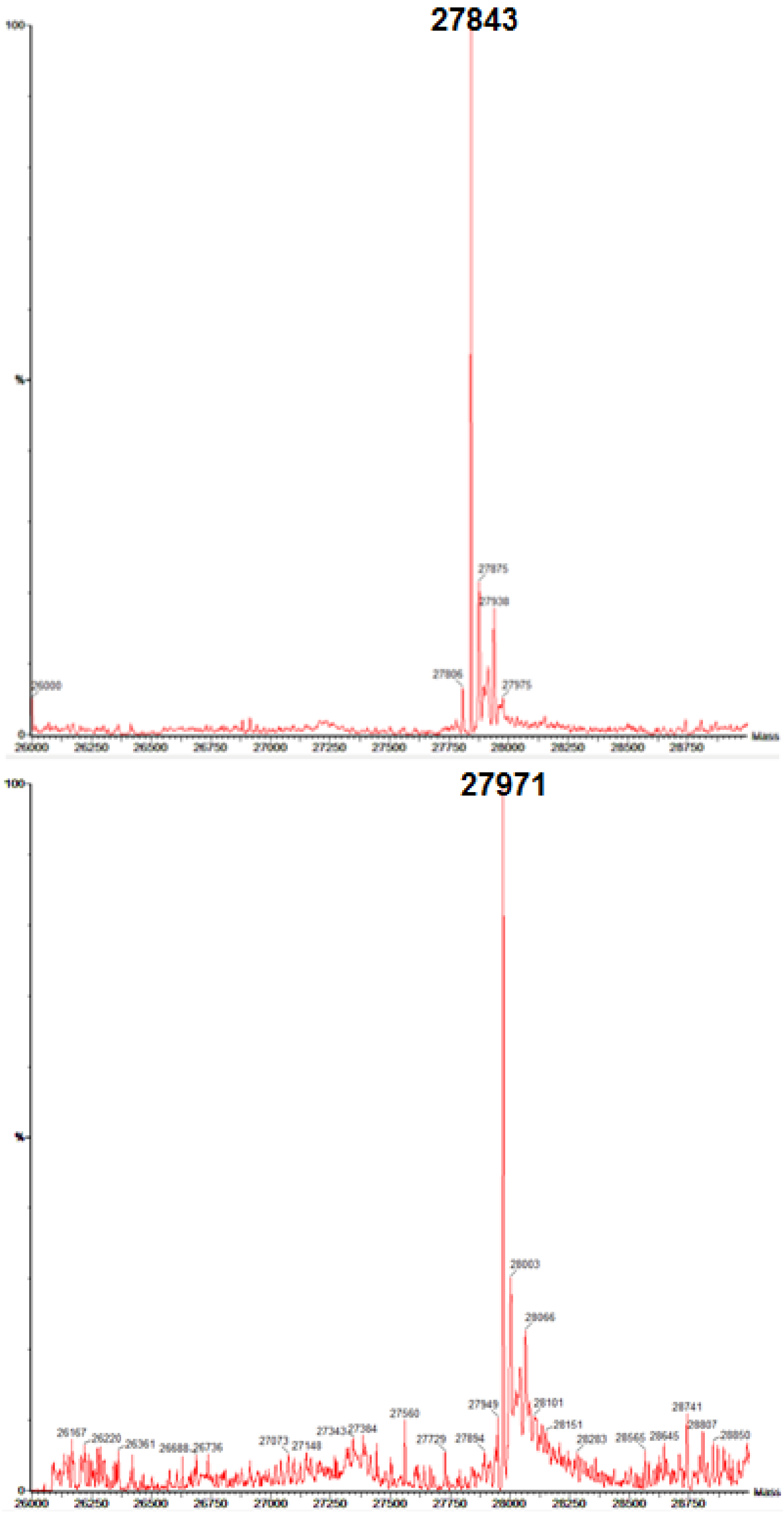

Fig. S6. MS spectra of unmodified Y58C SPM-1 (upper spectrum) and BTFA-labeled Y58C SPM-1 (lower spectrum). The observed mass difference corresponds to attachment of a single CH_2COCF_3-label per SPM-1 Y58C positive ion. Spectra were acquired using a Waters LCT Premier instrument fitted with a ToF analyzer. The positive ion electrospray ionization mode was used.

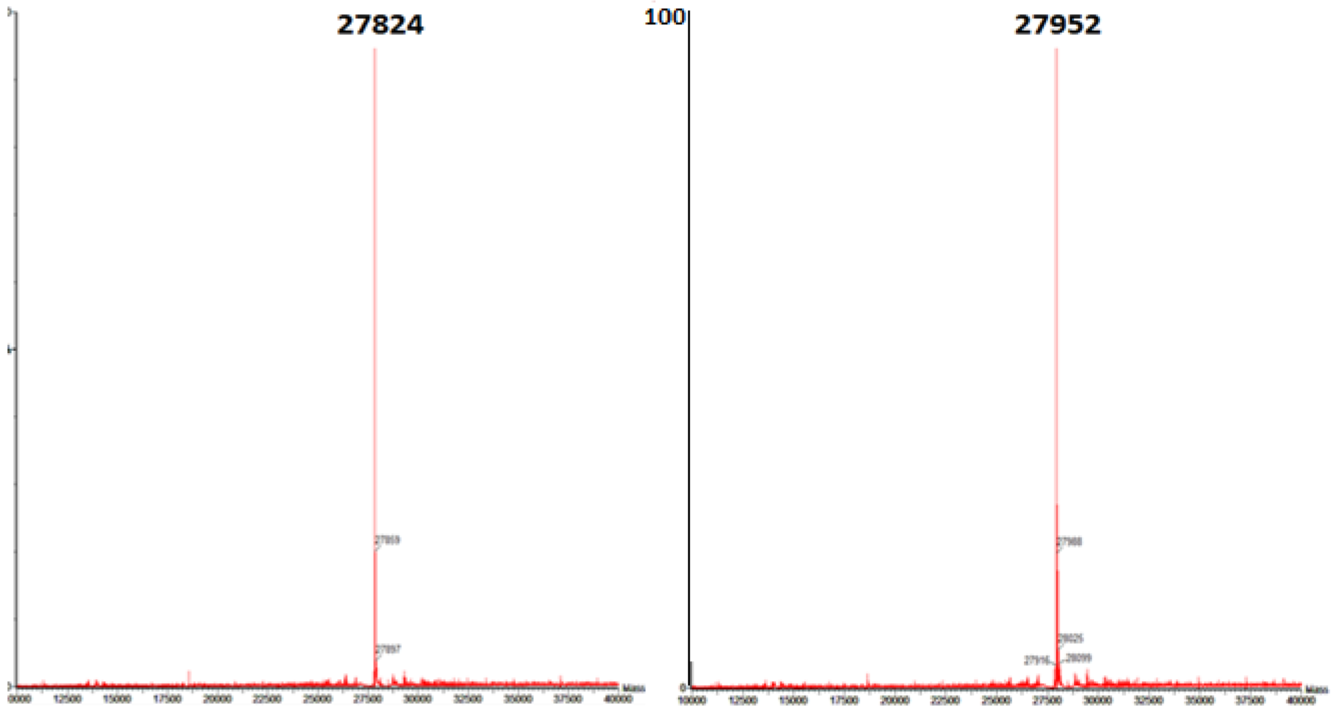

Fig. S7. MS spectra of unmodified F151C SPM-1 (left) and BTFA-labeled F151C SPM-1 (right). The observed mass difference corresponds to attachment of a single CH_2COCF_3-group per SPM-1 Y58C positive ion. Spectra were acquired using a Waters LCT Premier instrument fitted with a ToF analyzer. The positive ion electrospray ionization mode was used.

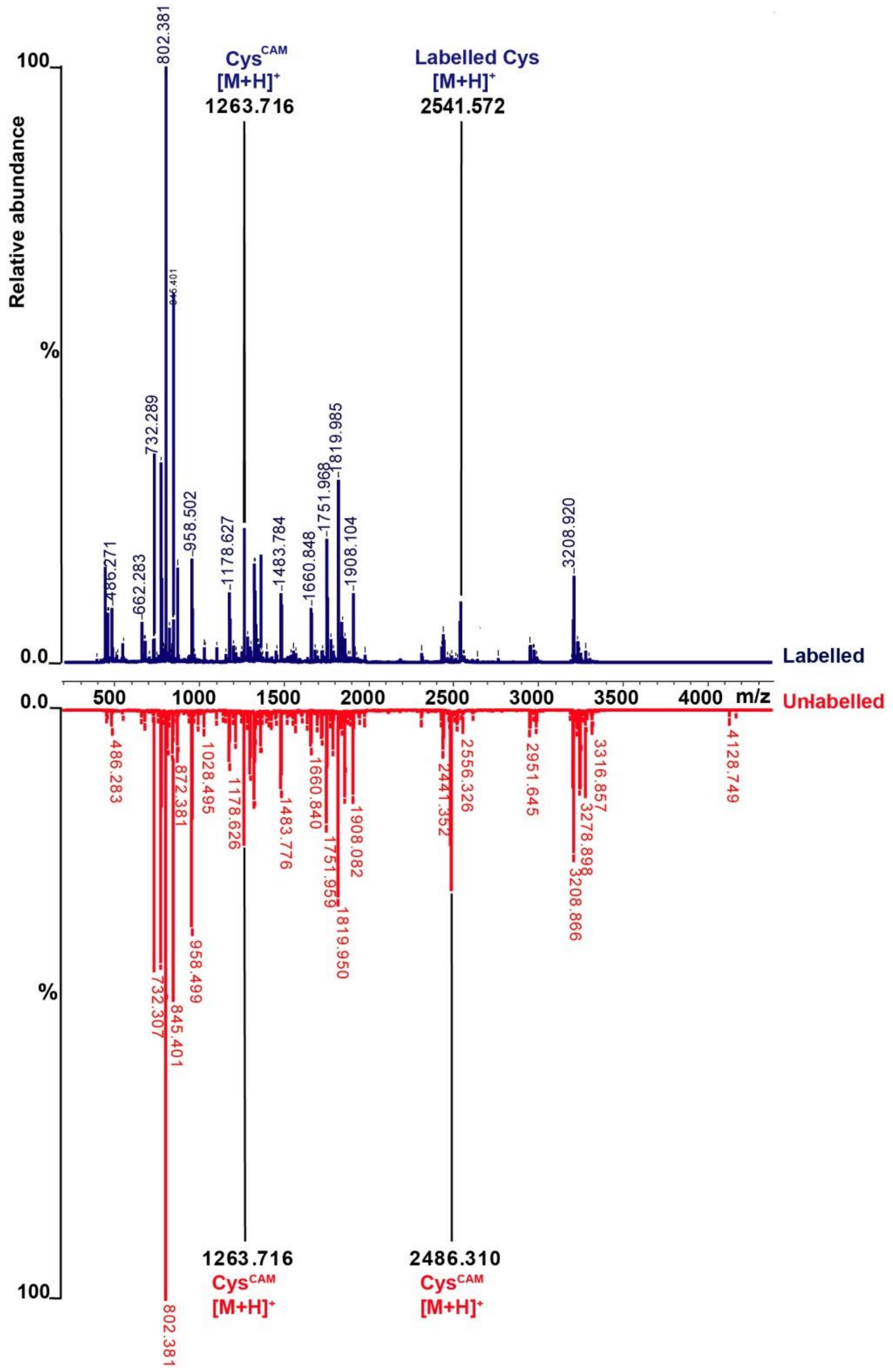

Fig. S8. MALDI-ToF MS spectra of BTFA-labeled (blue) and unlabeled (red) Y58C SPM-1 after digestion with porcine trypsin. Free cysteine in the sample was reduced (DTT) and *S*-carbamidomethylated (Cys^{CAM}) before digestion. A complete 'overview' of the digested sample is shown. Note that cysteine residue at the active site (Cys221) is unlabeled in both samples (m/z = 1263.716), whilst the targeted cysteine (Cys58) is labeled (m/z = 2541.572) in the labeled sample and is unlabeled (*S*-cabamidomethylated) in the control (m/z = 2486.310). <u>Calculation</u>: [2541.572 - (2486.310 – 57)] = 112±2 Da (practical mass corresponding to the incorporation of a CH_2COCF_3-label).

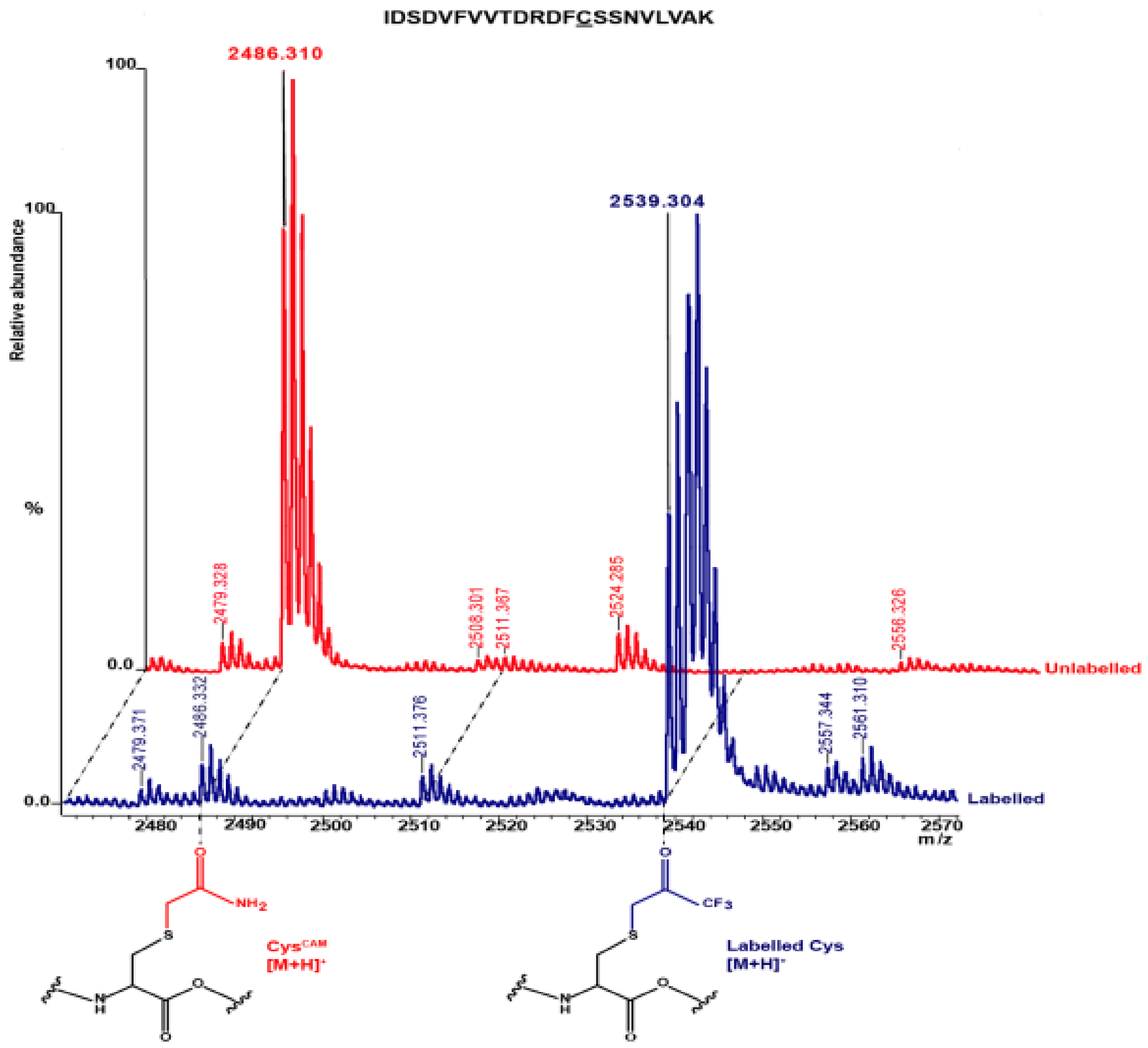

Fig. S9. MALDI-ToF MS spectra of BTFA-labeled (blue) and unlabeled (red) Y58C SPM-1 after digestion with porcine trypsin (close-up). Free cysteines were reduced (DTT) and *S*-carbamidomethylated (Cys^{CAM}) before digestion. Close-up view of the assigned modified peptide is shown (labeled: m/z = 2539.304, *S*-carbamidomethylated: m/z = 2486.3105). Cys58 was not fluorine-labeled in the control sample (red). The results imply the labeling procedure is efficient (blue).

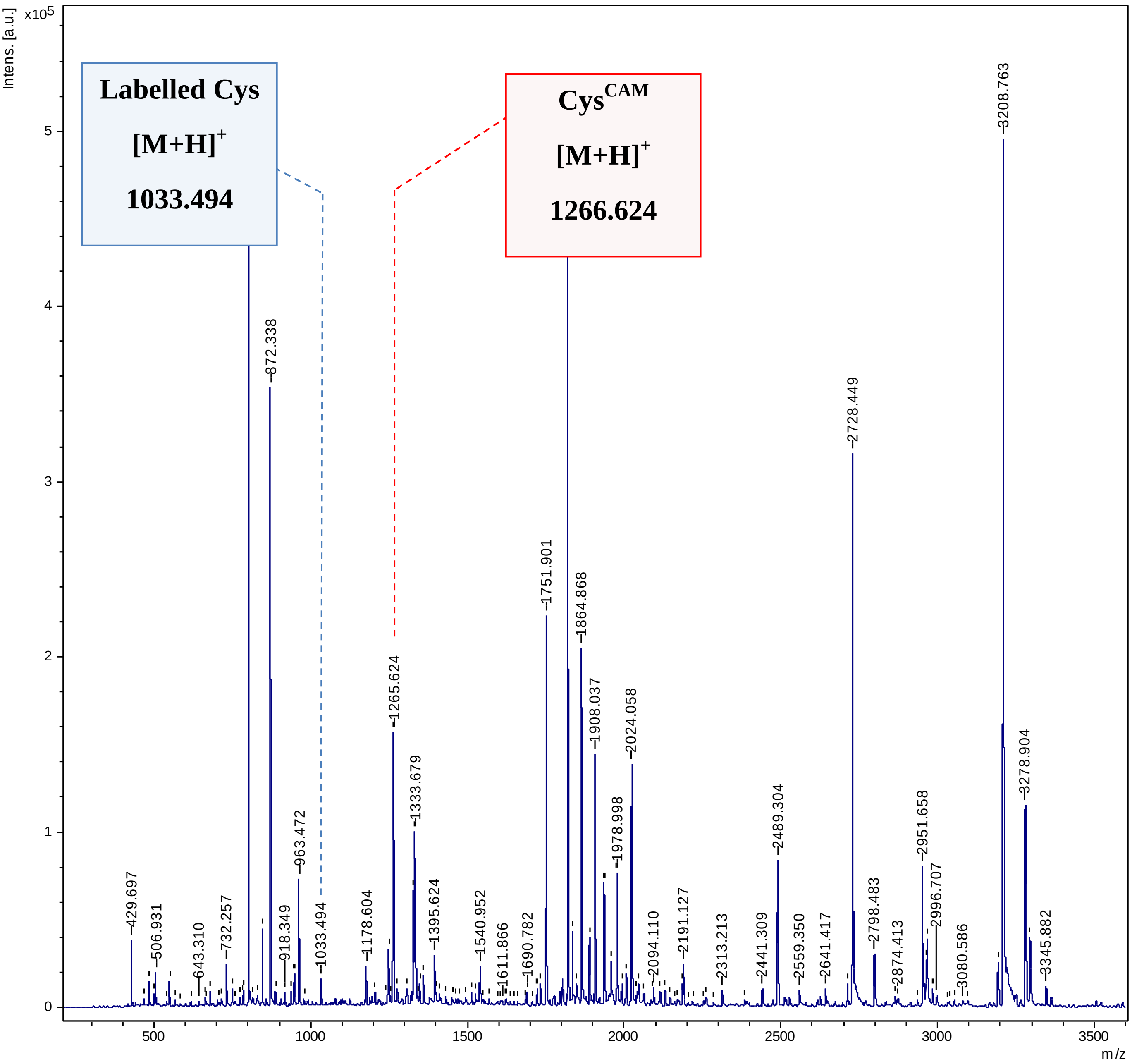

Fig. S10. MALDI-ToF MS spectrum of the BTFA-labeled F151C SPM-1 variant after digestion with porcine trypsin. The sequence of the peptidic fragment containing Cys221 is LLFGGCMIKPK (m/z = 1206) and that of the peptidic fragment containing Cys151 is IKAAECYK (m/z = 923). The sample was reduced (DTT) and *S*-carbamidomethylated (Cys^{CAM}) before digestion. A complete 'overview' of the digested sample is shown. The cysteine of the active site is unlabeled (*S*-carbamidomethylated, m/z = 1265.624) while the cysteine of interest (Cys151) was labeled efficiently (m/z = 1033.494).

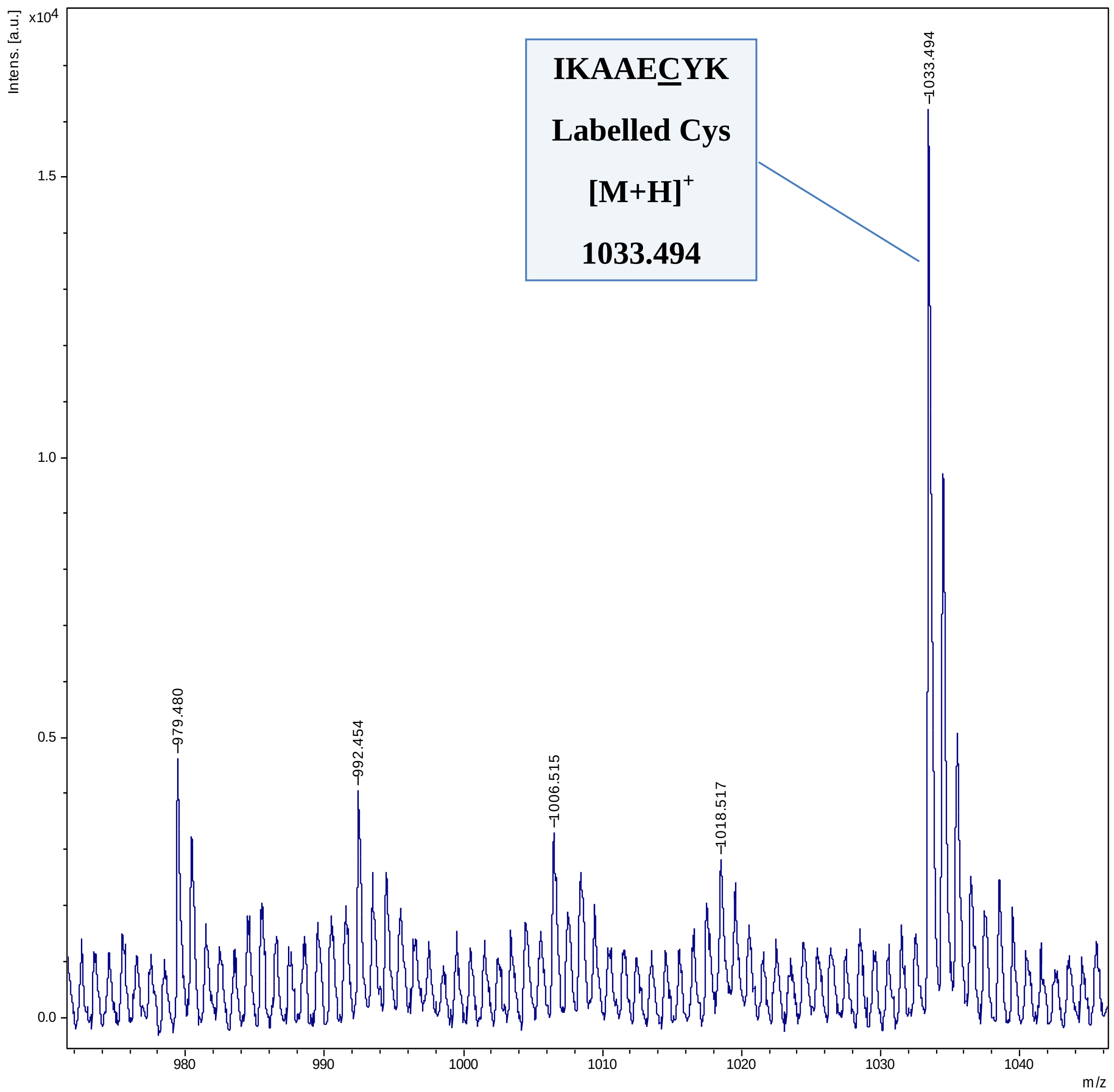

Fig. S11. MALDI-ToF MS spectra of BTFA-labeled F151C SPM-1 variant after digestion with porcine trypsin (close-up). The sample was reduced (DTT) and *S*-carbamidomethylated (Cys^{CAM}) before digestion. Close-up view of the assigned modified peptide (labelled: m/z = 1033.494). The observed mass shift corresponds to the incorporation of a CH_2COCF_3 group; the results imply the labeling procedure is efficient.

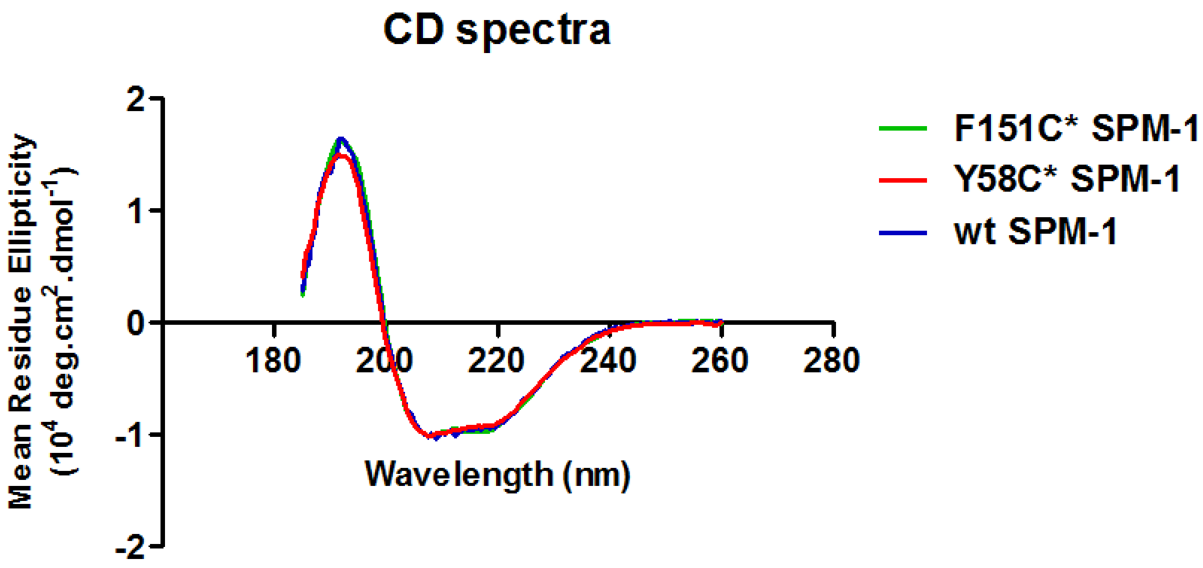

Fig. S12. CD spectra of SPM-1 variants. The CD spectra of wildtype SPM-1, Y58C*, and F151C* overlay quite-well, indicating that the secondary content of the proteins tested is comparable. Mean Residue Ellipticity was calculated using the equation:

$$\theta\ MRE(degcm^2\ dmol^{-1}) = \frac{\theta(mdeg)}{10 * Pathlength(cm) * [Protein](M) * n\ (no.\ of\ residues)}$$

.

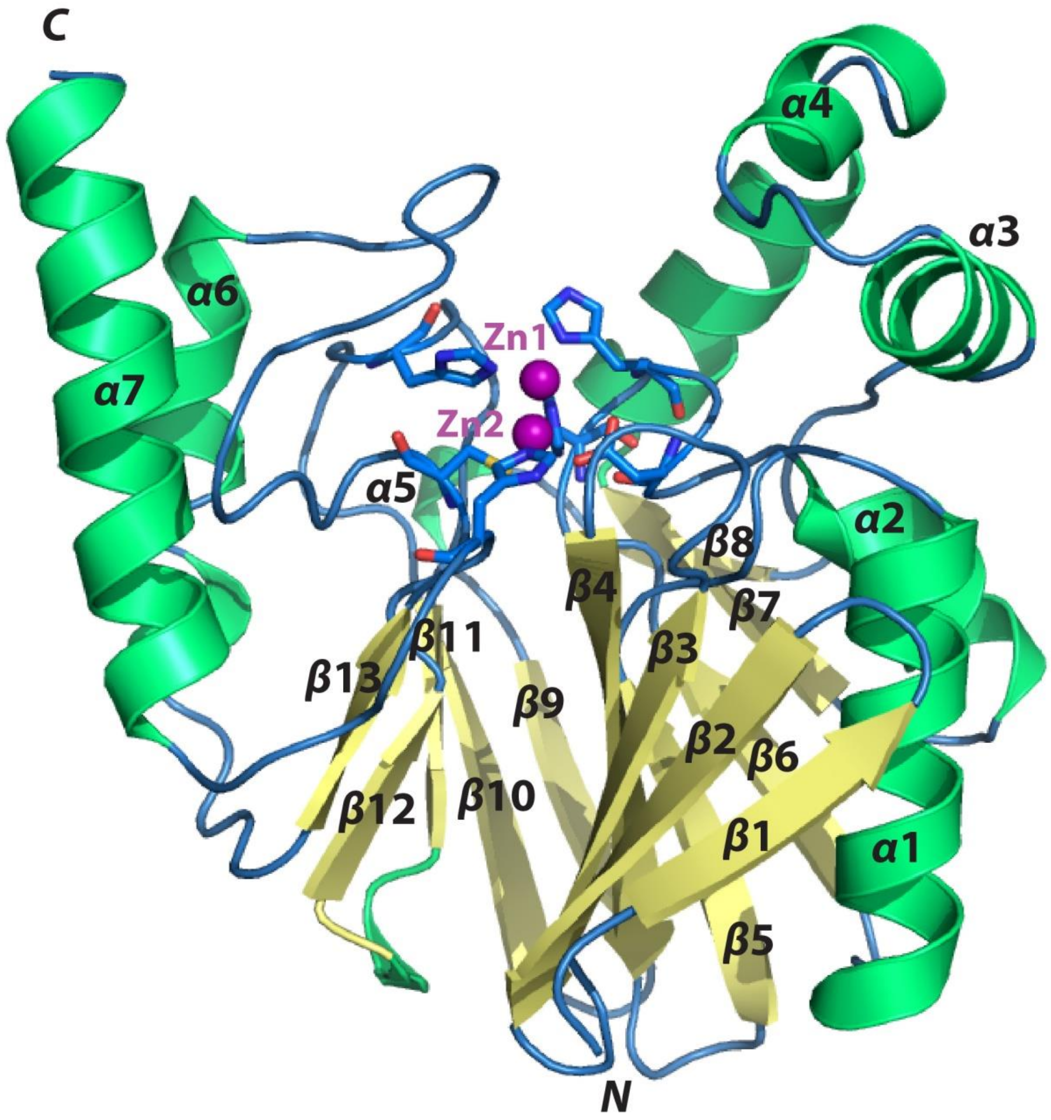

Fig. S13. View from the Y58C SPM-1 variant crystal structure. (PDB ID: 5LS3). The obtained crystal structure shows that Y58C SPM-1 has the conserved αββα MBL fold.[19]

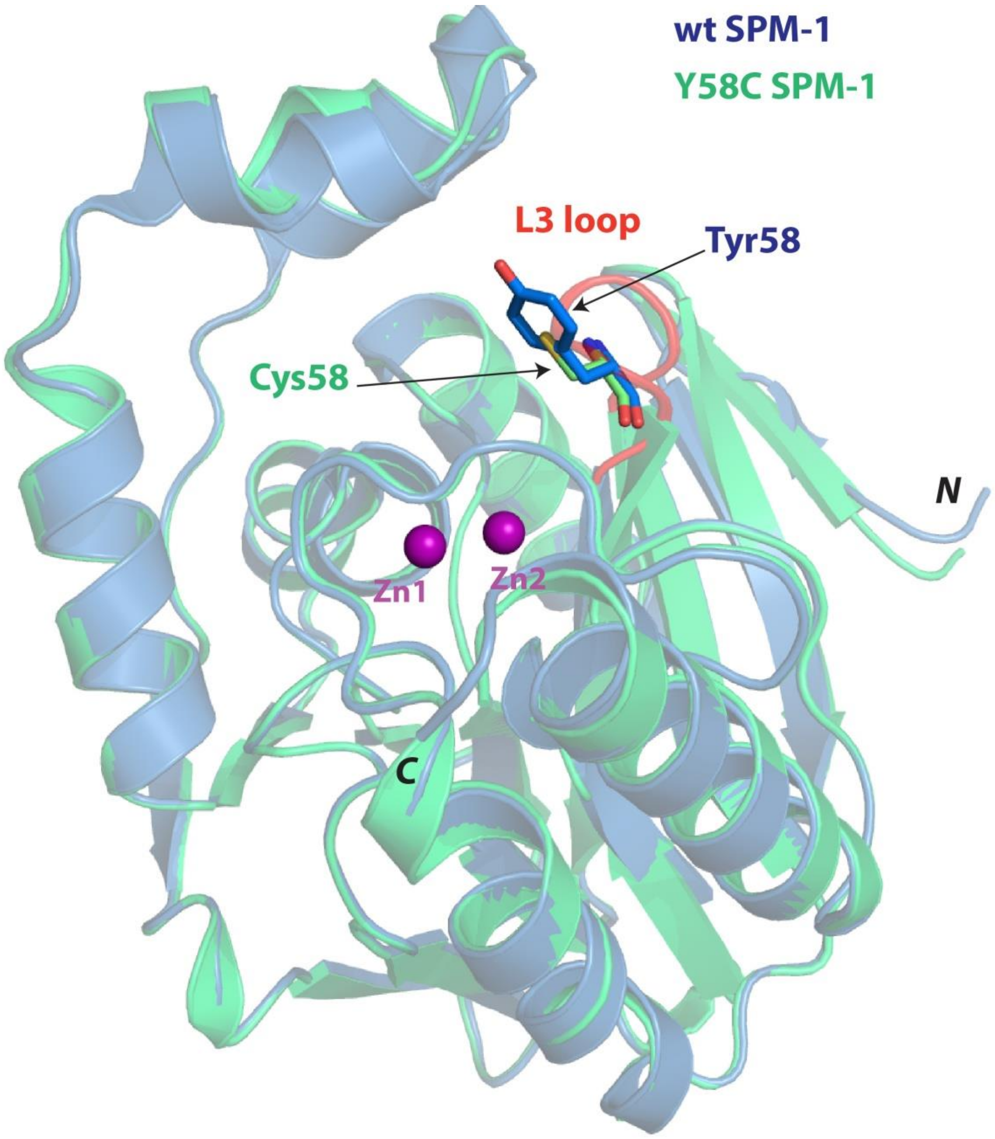

Fig. S14. Overlay of wildtype SPM-1 and Y58C SPM-1 variant crystal structures. Superimposition shows that Y58 (in wt SPM-1 structure, PDB ID: 4BP0) and C58 (in Y58C SPM-1, PDB ID: 5LS3) have the same orientations on the L3 loop.[18]

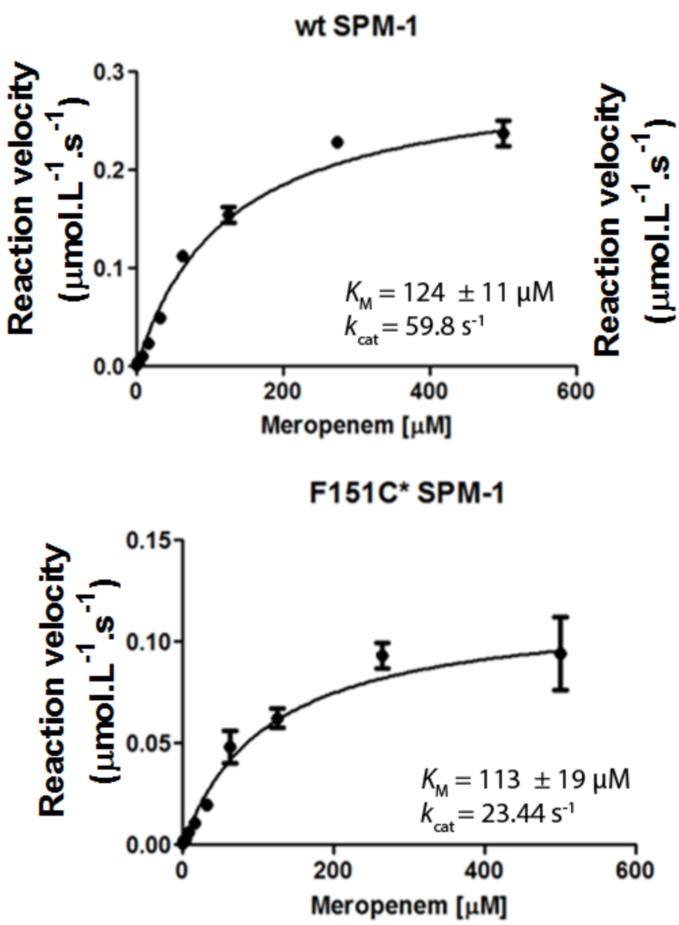

Fig. S15. Meropenem turnover by wt SPM-1, Y58C* SPM-1, and F151C* SPM-1. Error bars denote SEM, n = 3. Curves were fitted using GraphPad Prism.

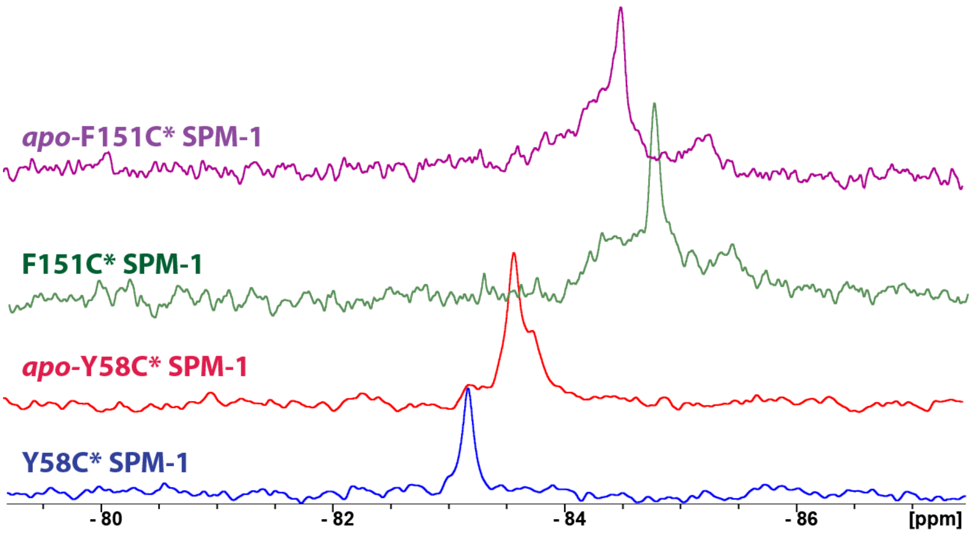

Fig. S16. PrOF NMR spectra of *apo*- and di-Zn(II)-SPM-1* variants. The assay mixtures contained SPM-1* variant (40 µM) in 50 mM Tris, pH 7.5, in 90 % H_2O and 10 % D_2O.

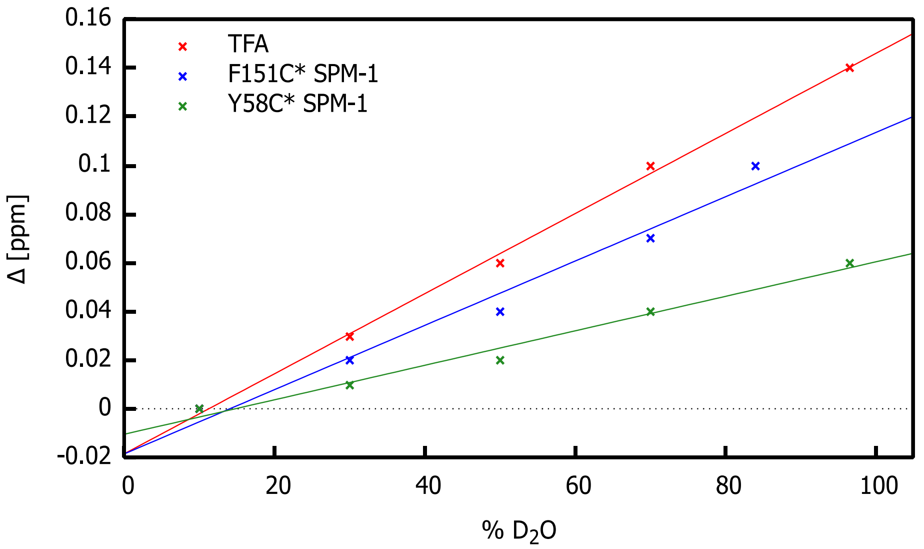

Fig. S17. Solvent exposure studies of SPM-1* variants by PrOF NMR. Plot of the chemical shift difference ($\Delta = \delta_{obs} - \delta_0$) of the protein peak against % D_2O (in H_2O) content. δ_0 refers to the chemical shift observed with 10% D_2O. Linear regression analyses revealed fitting functions of: y = 0.00132x-0.018 for F151C* SPM-1; y = 0.00071x-0.010 for Y58C* SPM-1; y = 0.00164x-0.018 for TFA. The results imply that the F151C* label is more exposed to solvent than the Y58C* label (see Fig. S2B).

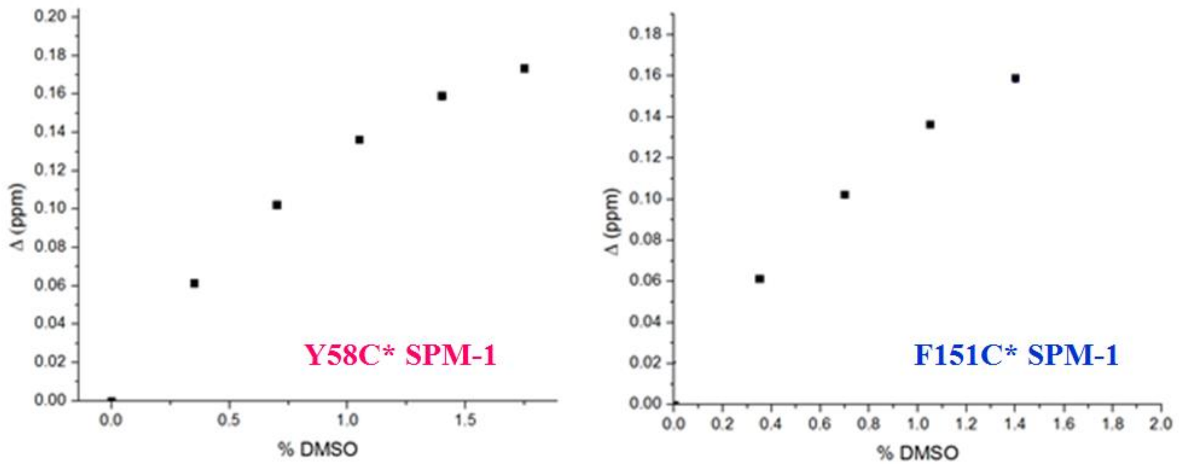

Fig. S18. DMSO titration studies with SPM-1* variants by PrOF NMR. The assay mixtures contained the SPM-1* variant (40 μM) in 50 mM Tris, pH 7.5, in 90 % H_2O and 10 % D_2O. Samples contained 0.8 % DMSO at the highest ligand concentration (400 μM).

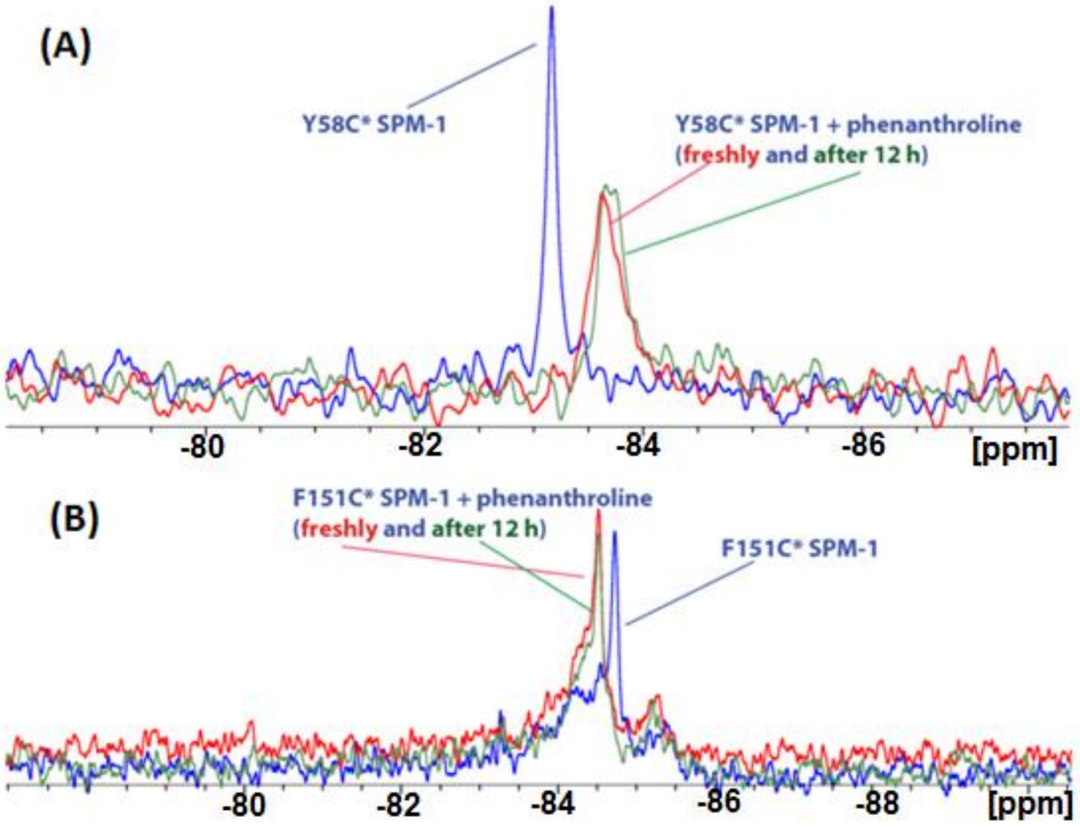

Fig. S19. ^{19}F-NMR time-course spectra of SPM-1* variants treated with 1,10-*o*-phenanthroline. The assay mixtures contained 40 µM Y58C* SPM-1 **(A)** (blue trace) or F151C* SPM-1 **(B)** and 400 µM ligand (freshly: red trace, after 12 hours: green) in 50 mM Tris, pH 7.5, in 90 % H_2O and 10 % D_2O.

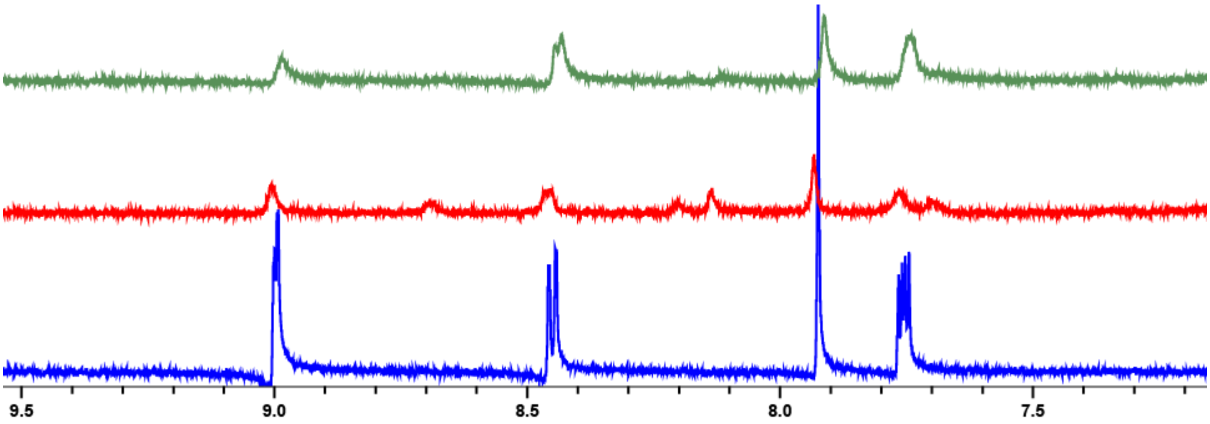

Fig. S20. ^{1}H CPMG NMR spectra of 1,10-*o*-phenanthroline treated with SPM-1* variants. The assay mixture contained 400 µM ligand (blue trace), in the presence of 40 µM Y58C* SPM-1 (red trace) or 40 µM F151C* SPM-1 (green trace) after monitoring the reaction for 10 min in 50 mM Tris-D_{11}, pH 7.5, in 90 % H_2O and 10 % D_2O.

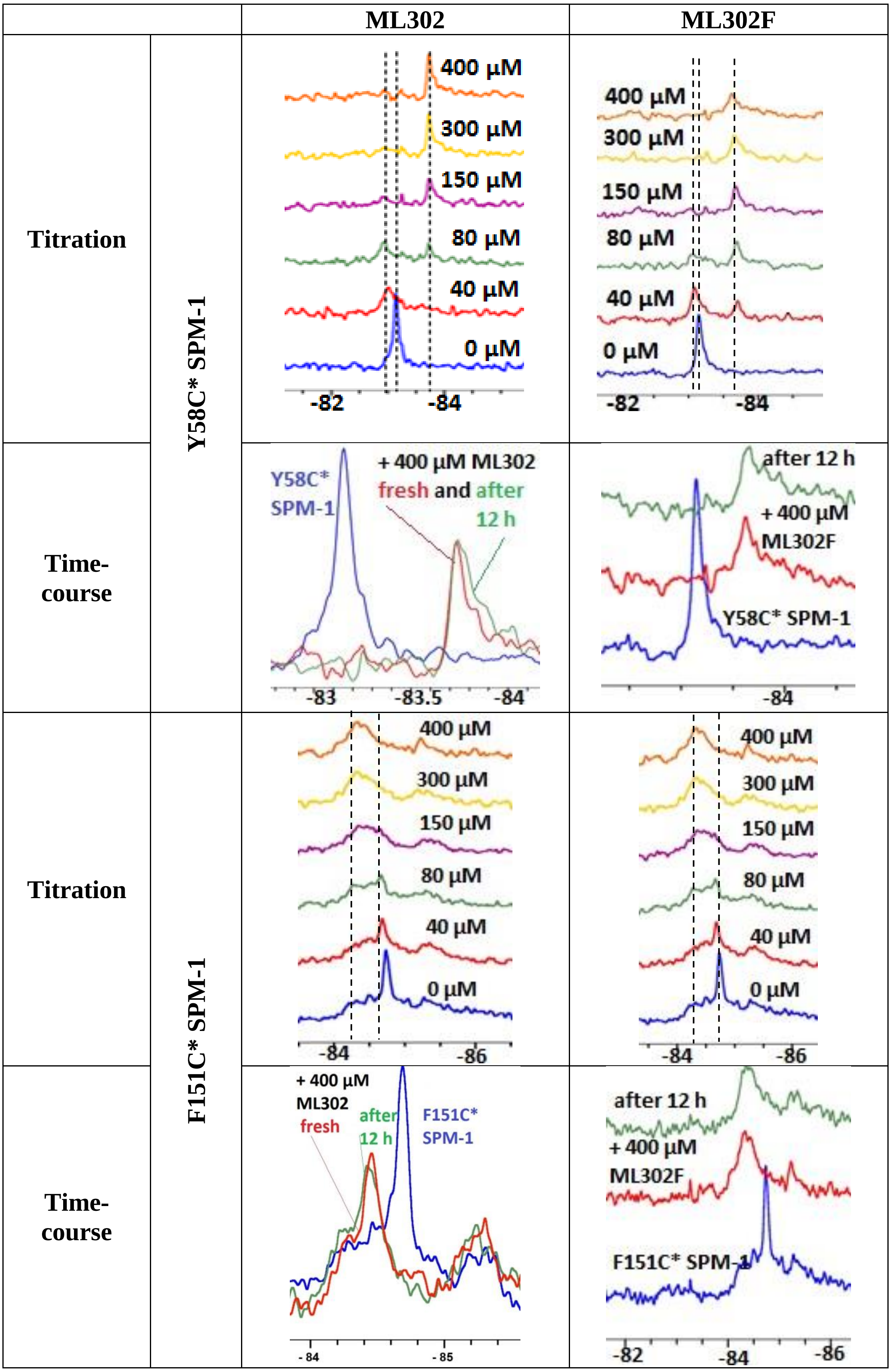

Fig. S21. PrOF NMR analyses of ML302 and ML302F and SPM-1* variants. The assay mixtures contained 40 µM SPM-1* and the added ligand in 50 mM Tris, pH 7.5, in 90 % H_2O and 10 % D_2O.

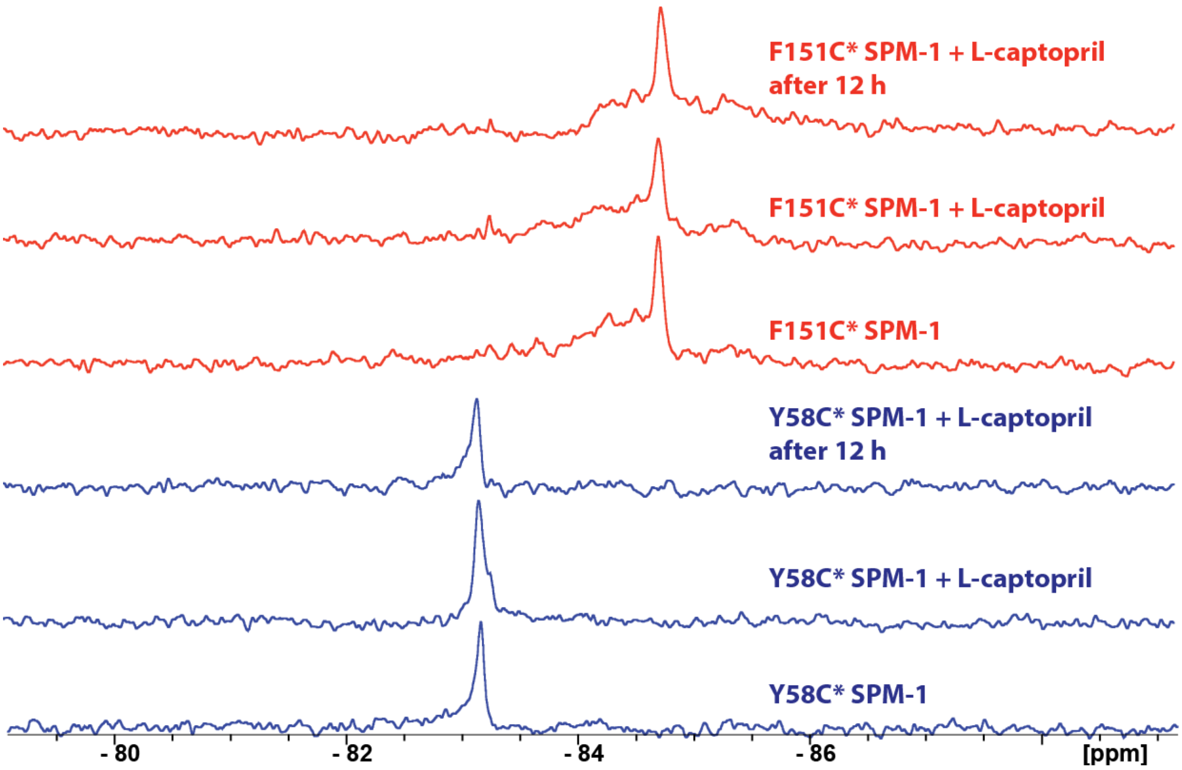

Fig. S22. ^{19}F-NMR spectra of L-captopril and SPM-1* solutions. No substantial changes are observed upon addition of 400 µM L-captopril to SPM-1* solutions, including after 12 h. The assay mixtures contained 40 µM SPM-1* in 50 mM Tris, pH 7.5, in 90 % H_2O and 10 % D_2O.

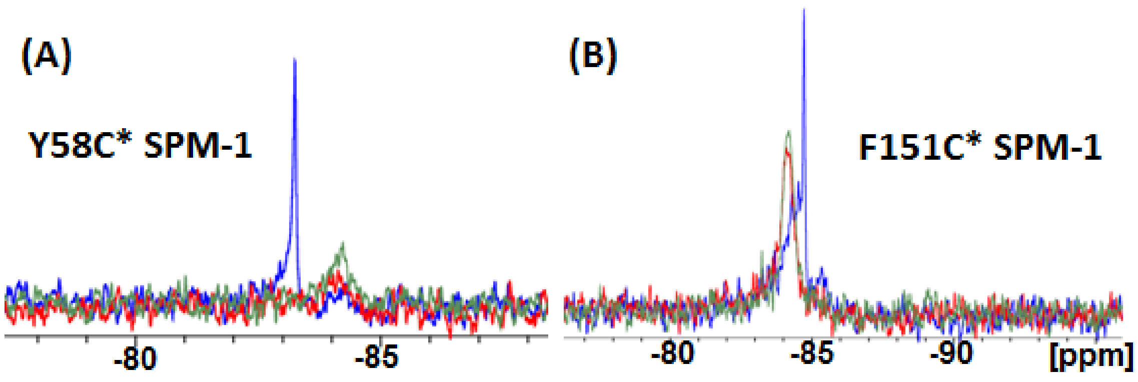

Fig. S23. ^{19}F-NMR time-course spectra of SPM-1* variants with the isoquinoline derivative (1). The assay mixtures contained 40 µM Y58C* SPM-1 **(A)** (blue trace) or F151C* SPM-1 **(B)** and 400 µM isoquinoline derivative **(1)** (freshly mixed: red trace, after 12 hours: green) in 50 mM Tris, pH 7.5, in 90 % H_2O and 10 % D_2O.

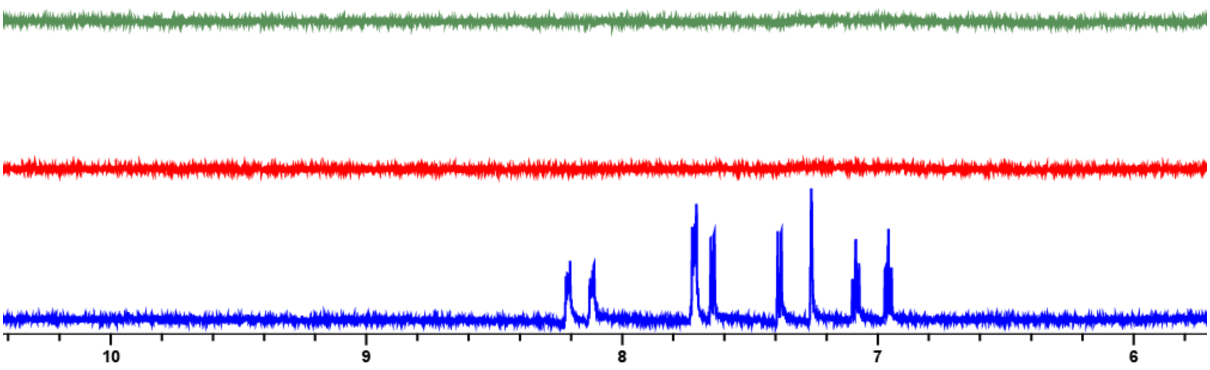

Fig. S24. ^{1}H CPMG NMR spectra of the isoquinoline derivative (1) with SPM-1* variants. The assay mixture contained 400 µM ligand (blue trace), in the presence of 40 µM Y58C* SPM-1 (red trace) or in the presence of 40 µM F151C* SPM-1 (green trace) after monitoring the reaction for 10 min in 50 mM Tris-D_{11}, pH 7.5, in 90 % H_2O and 10 % D_2O.

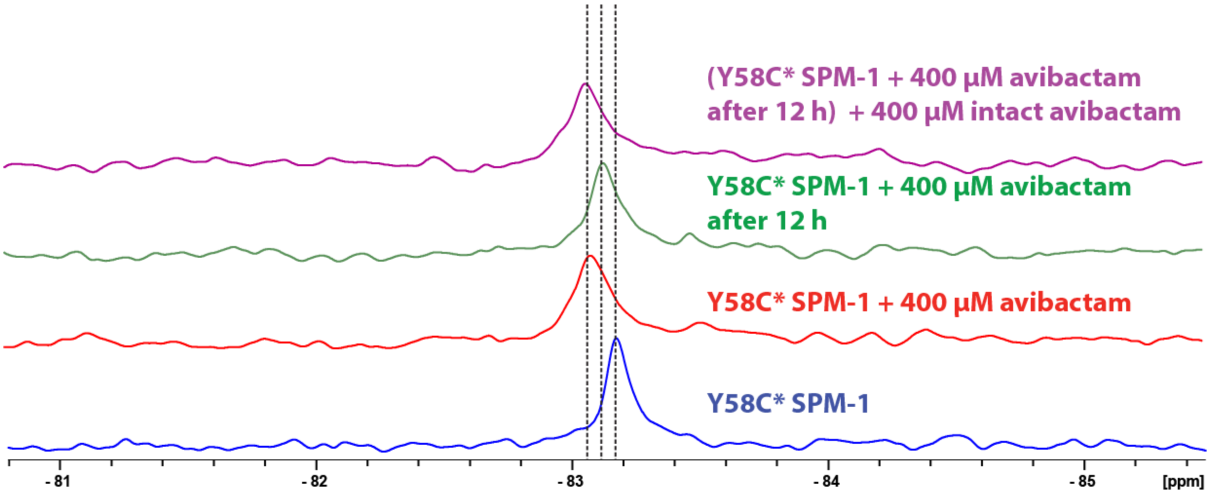

Fig. S25. ^{19}F-NMR spectra of avibactam and Y58C* SPM-1. The assay mixtures contained 40 µM SPM-1* in 50 mM Tris, pH 7.5, in 90 % H_2O and 10 % D_2O.

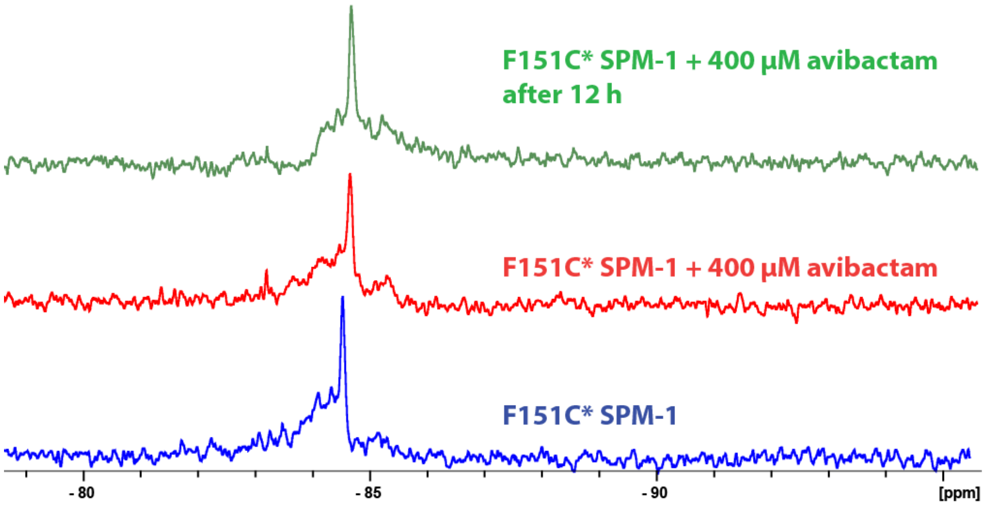

Fig. S26. ^{19}F-NMR spectra of avibactam and F151C* SPM-1. The assay mixtures contained 40 µM SPM-1* in 50 mM Tris, pH 7.5, in 90 % H_2O and 10 % D_2O.

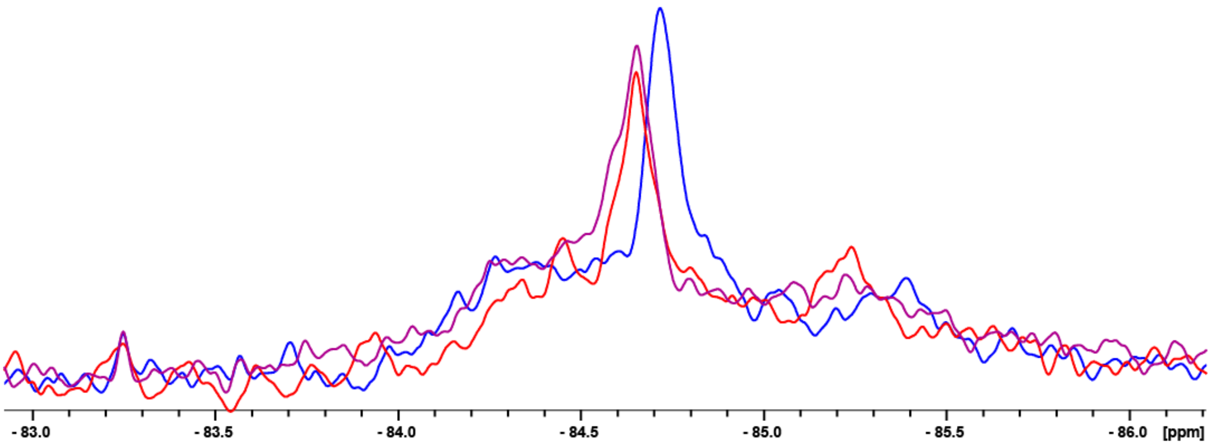

Fig. S27. ^{19}F-NMR time-course spectra of F151C* SPM-1 with meropenem. The assay mixtures contained 40 µM F151C* SPM-1 (blue trace) and 400 µM ligand (freshly: red trace, after 12 hours: purple) in 50 mM Tris, pH 7.5, in 90 % H_2O and 10 % D_2O.

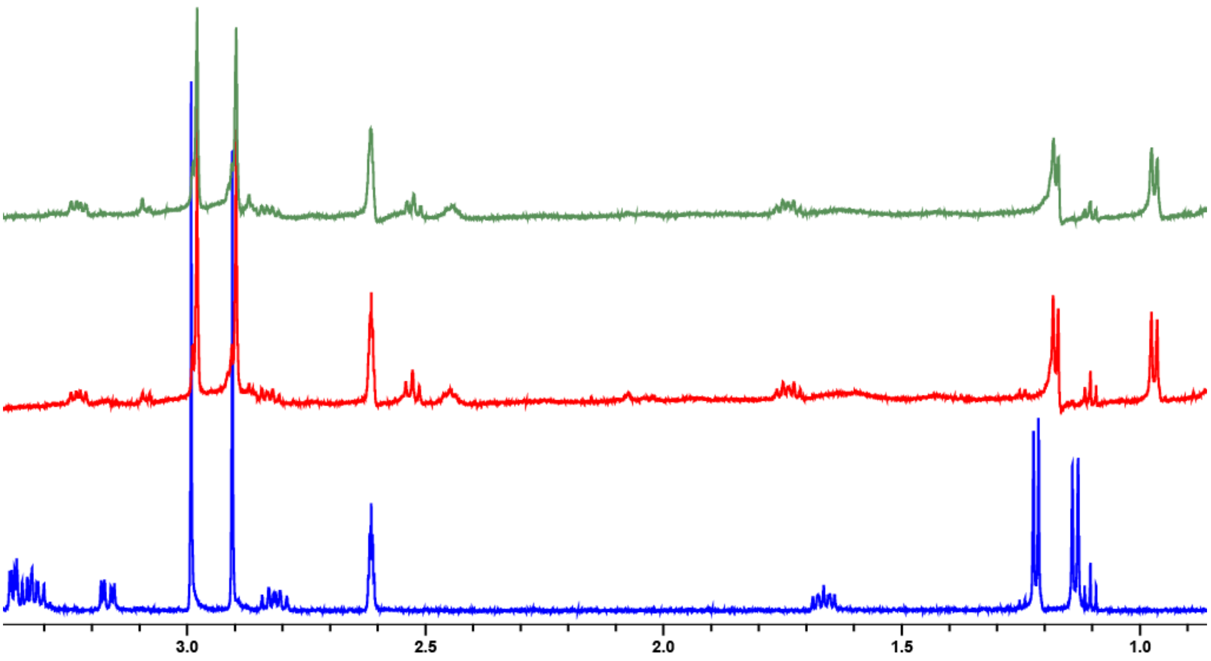

Fig. S28. ^{1}H CPMG NMR spectra of meropenem with SPM-1 variants. The assay mixture contained 400 µM meropenem (blue trace), in the presence of 40 µM Y58C* SPM-1 (red trace) or 40 µM F151C* SPM-1 (green trace) after monitoring the reaction for 10 min in 50 mM Tris-D_{11}, pH 7.5, in 90 % H_2O and 10 % D_2O.

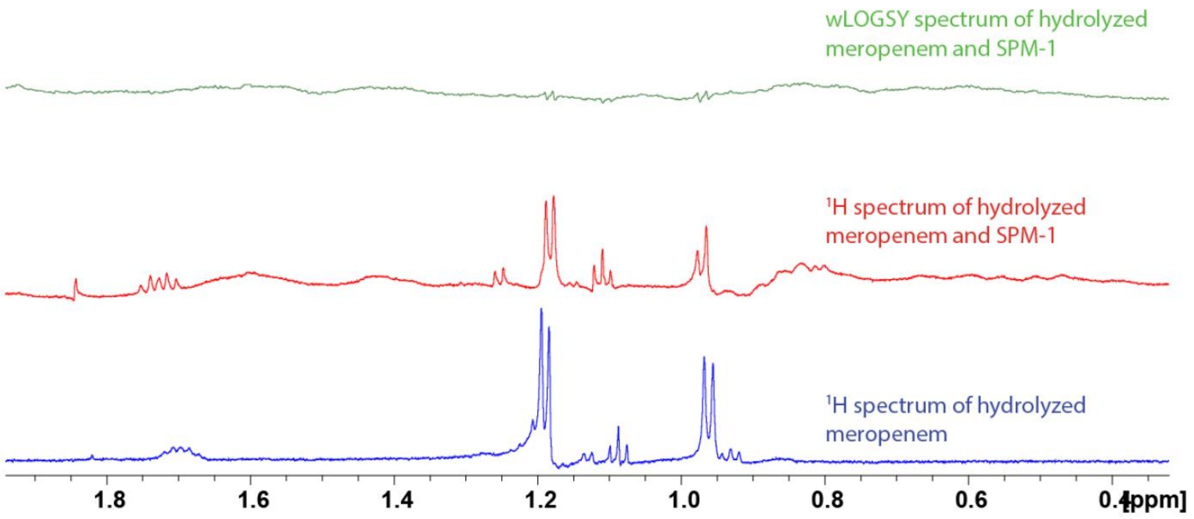

Fig. S29. Monitoring the binding of the products of meropenem hydrolysis to SPM-1 by ^{1}H and wLOGSY NMR. The assay mixture contained 400 µM hydrolyzed meropenem (by BcII) with 40 µM SPM-1 in 50 mM Tris-D_{11}, pH 7.5, in 90 % H_2O and 10 % D_2O.

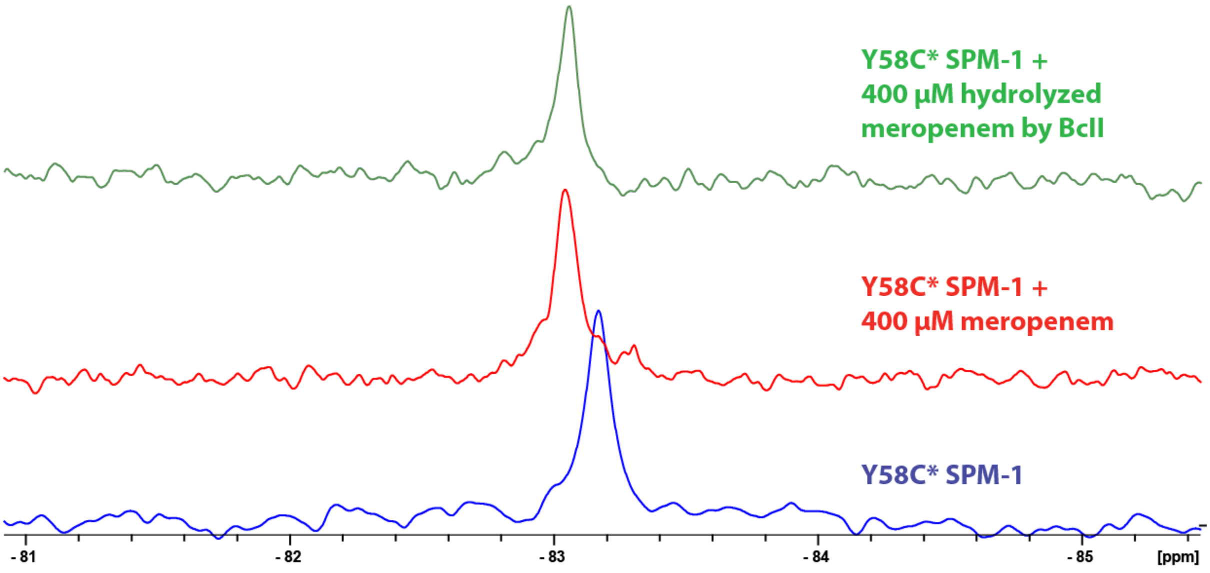

Fig. S30. ^{19}F-NMR of Y58C* SPM-1 interaction with hydrolyzed meropenem. The assay mixtures contained 40 µM Y58C* SPM-1 (blue trace) and 400 µM added ligand in 50 mM Tris, pH 7.5, in 90 % H_2O and 10 % D_2O.

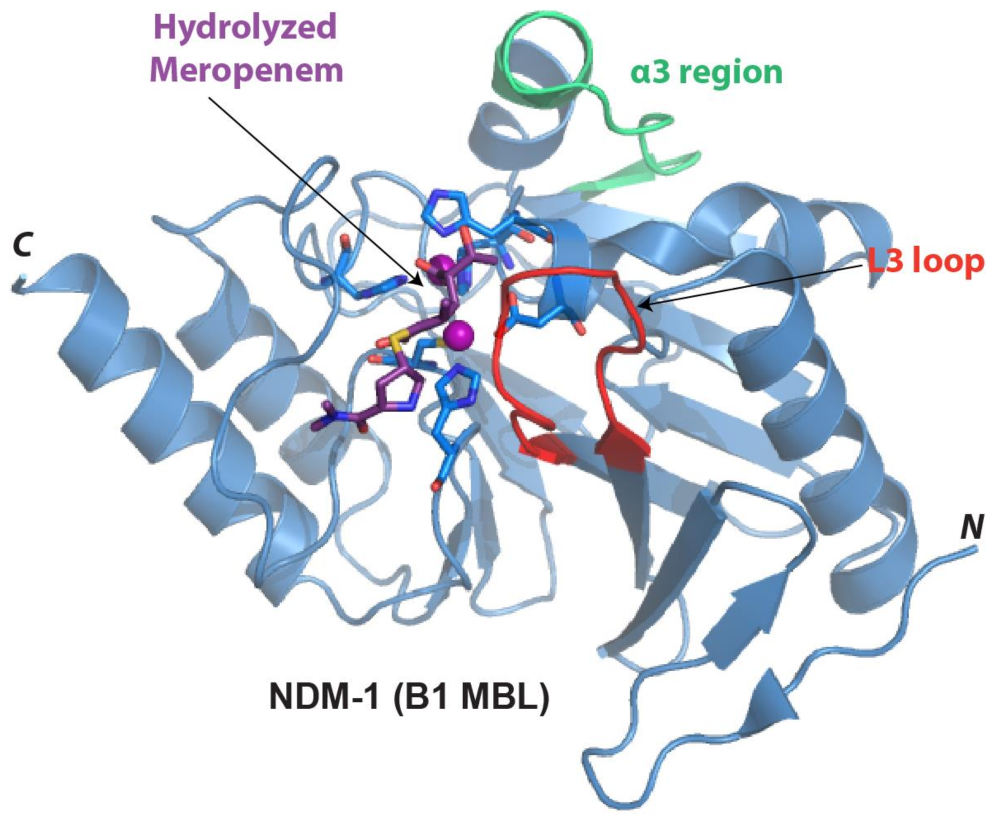

Fig. S31. View from NDM-1 crystal structure in complex with hydrolyzed meropenem. (PDB ID: 4EYL).[20] Hydrolyzed meropenem binds to the active site of New Delhi MBL-1 (NDM-1), a B1 subfamily MBL, in close proximity to the L3 loop.

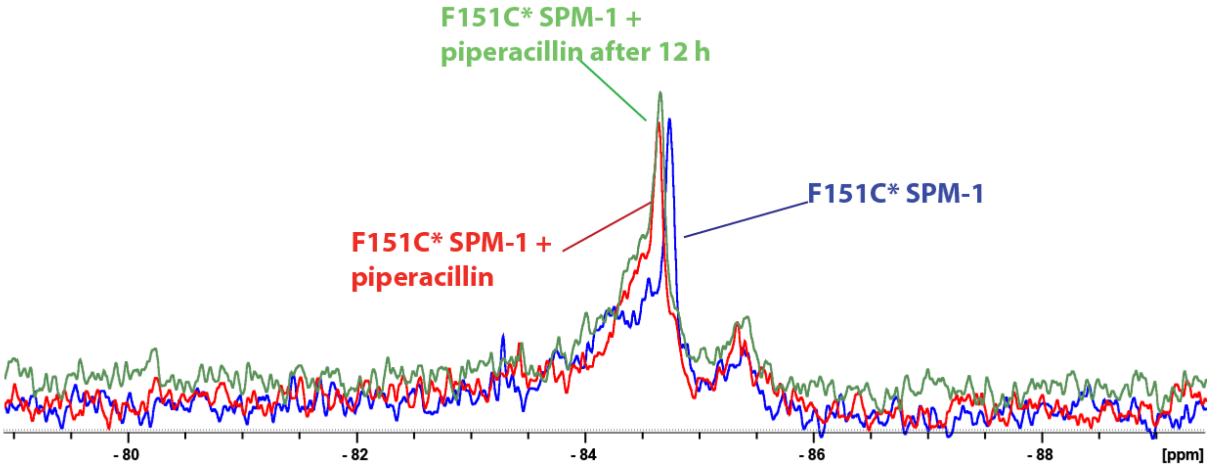

Fig. S32. ^{19}F-NMR time-course spectra of piperacillin with F151C* SPM-1. The assay mixtures contained 40 µM SPM-1* and 400 µM piperacillin in 50 mM Tris, pH 7.5, in 90 % H_2O and 10 % D_2O.

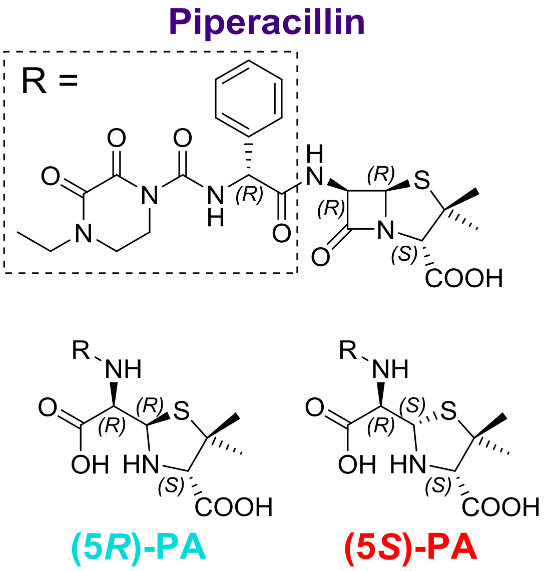

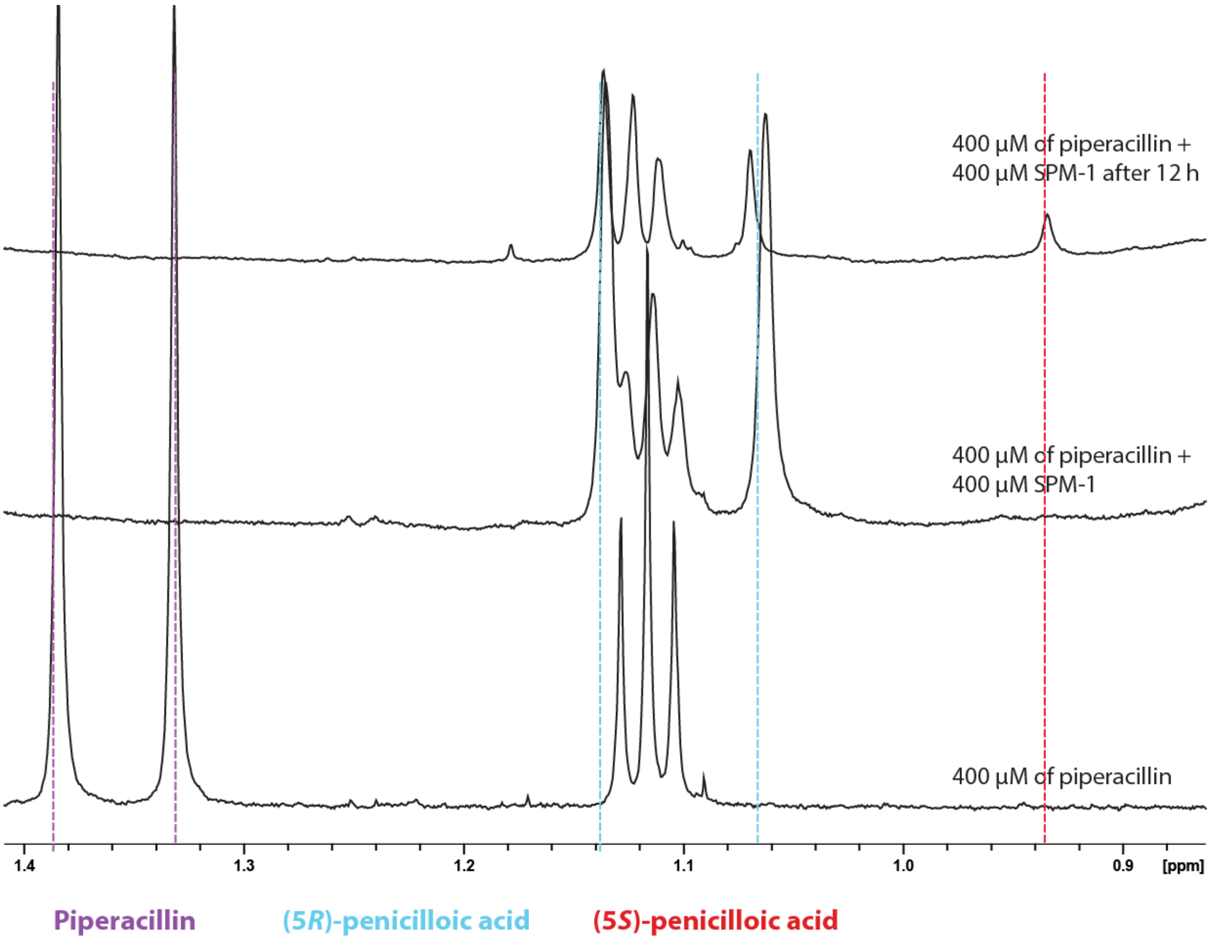

Fig. S33. Monitoring the reaction of piperacillin with SPM-1 after 12 h. The assay mixture contained 400 µM piperacillin with 40 µM SPM-1 in 50 mM Tris-D_{11}, pH 7.5, in 90 % H_2O and 10 % D_2O. Spectral assignments are reported by van Berkel *et al.*[21]

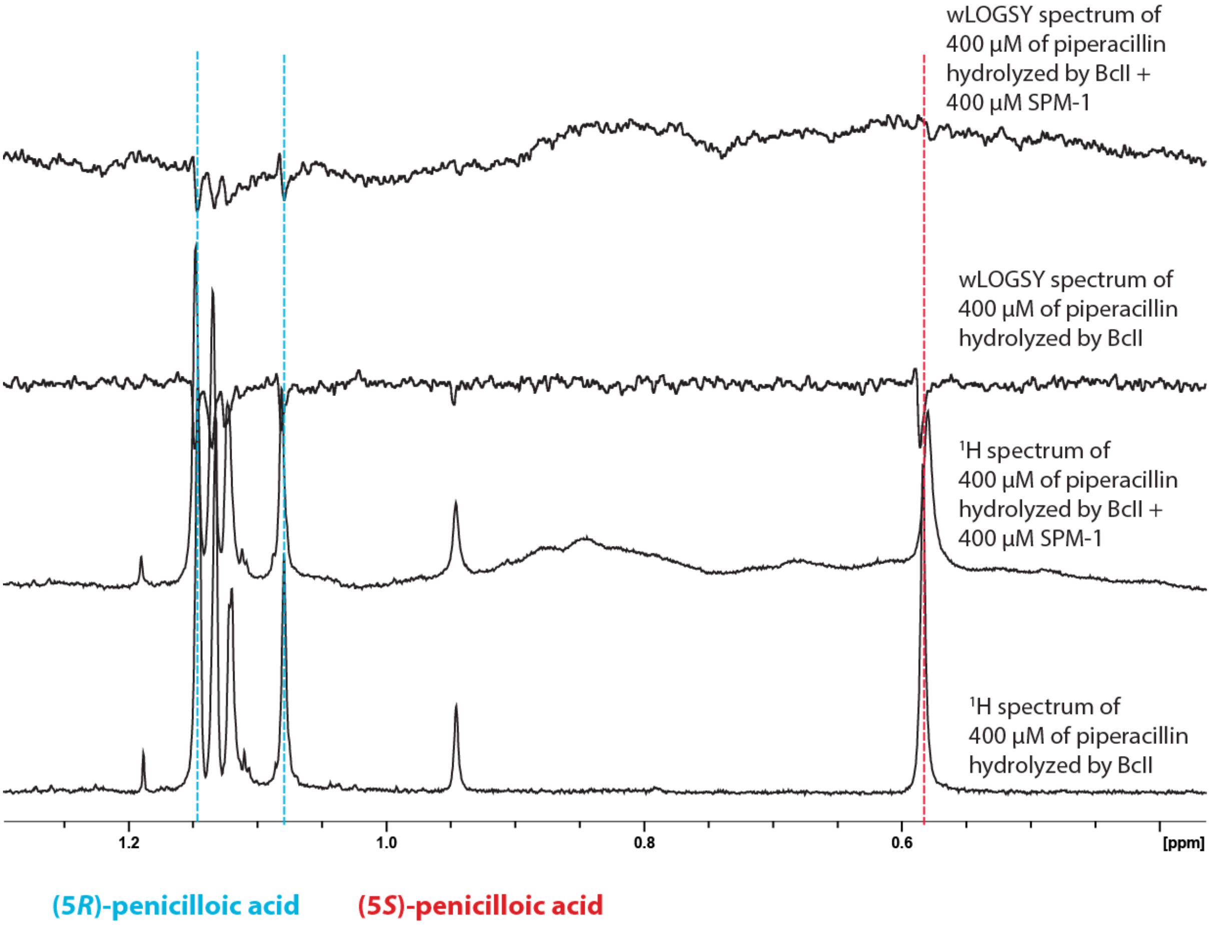

Fig. S34. Monitoring the binding of hydrolyzed piperacillin to SPM-1 by ^{1}H and wLOGSY NMR. The assay mixture contained 400 μM hydrolyzed piperacillin (by BcII)[21] with 40 μM SPM-1 in 50 mM Tris-D_{11}, pH 7.5, in 90 % H_2O and 10 % D_2O. Spectral assignments are reported by van Berkel *et al*.[21]

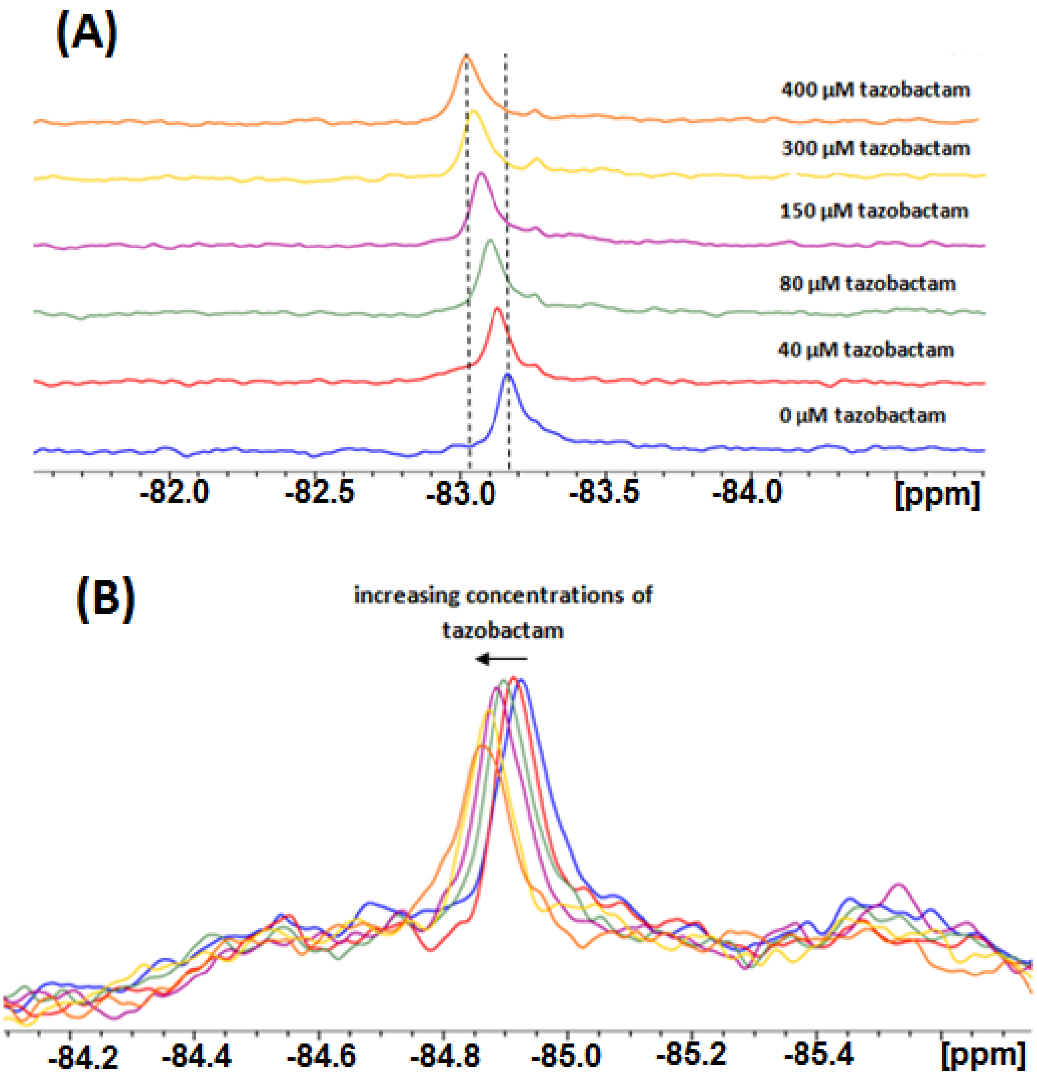

Fig. S35. ^{19}F-NMR spectra of the titration of tazobactam into Y58C* SPM-1 (A) and F151C* SPM-1 (B) solution. The assay mixtures, to which tazobactam was titrated, contained 40 µM SPM-1* in 50 mM Tris, pH 7.5, in 90 % H_2O and 10 % D_2O.

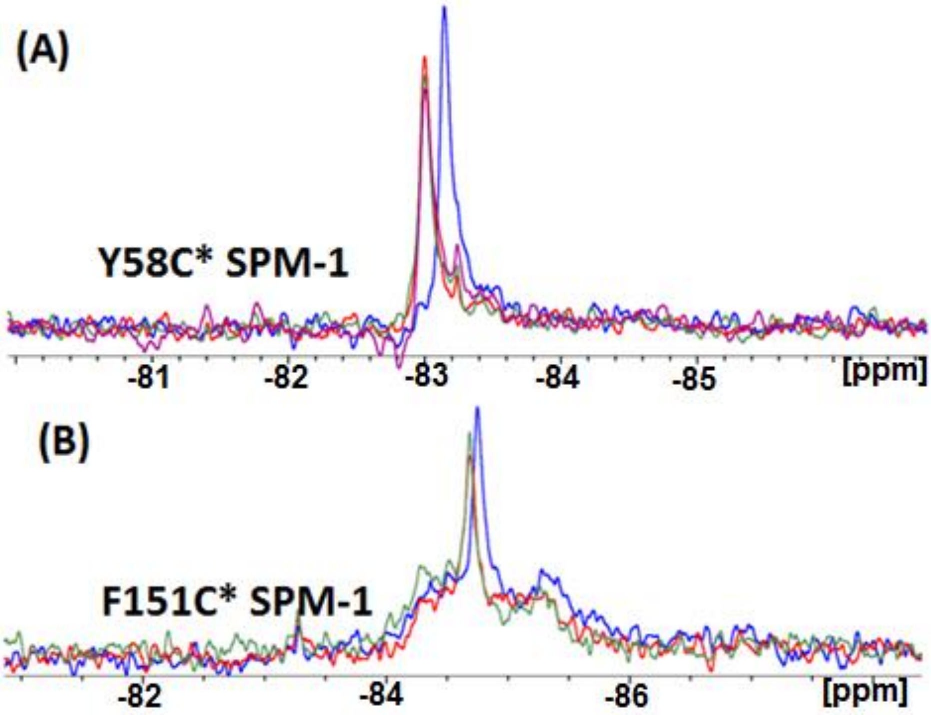

Fig. S36. ^{19}F-NMR time-course spectra of SPM-1* variants with tazobactam. The assay mixtures contained 40 µM Y58C* SPM-1 **(A)** or F151C* SPM-1 **(B)** (blue trace) and 400 µM tazobactam (freshly: red trace, after 12 hour in duplicate: green and purple traces) in 50 mM Tris, pH 7.5, in 90 % H_2O and 10 % D_2O.

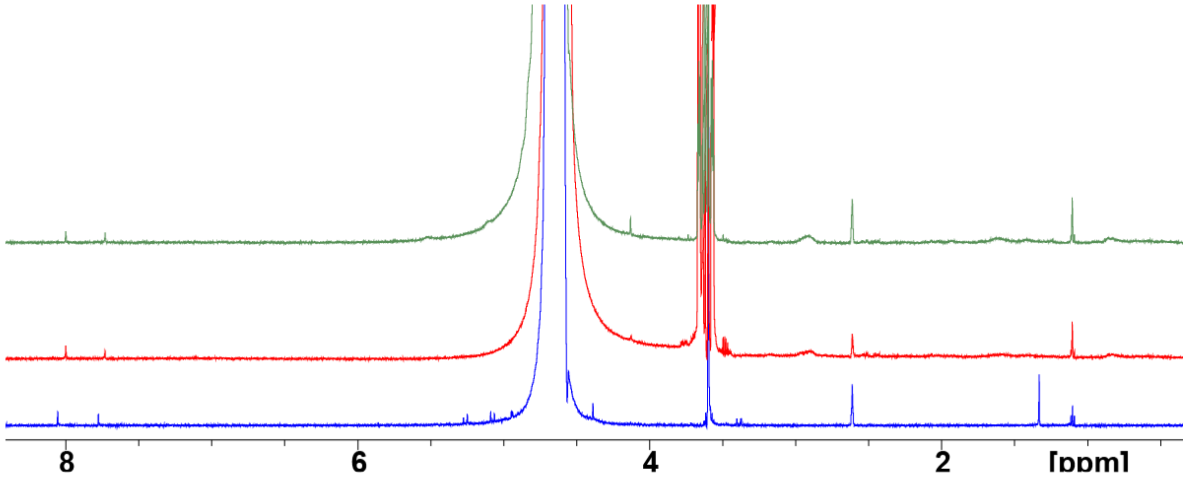

Fig. S37. ^{1}H CPMG NMR spectra of tazobactam with SPM-1* variants. The assay mixture contained 400 µM tazobactam (blue trace), in the presence of 40 µM Y58C* SPM-1 (red trace) or 40 µM F151C* SPM-1 (green trace) after monitoring the reaction for 10 min in 50 mM Tris-D_{11}, pH 7.5, in 90 % H_2O and 10 % D_2O.

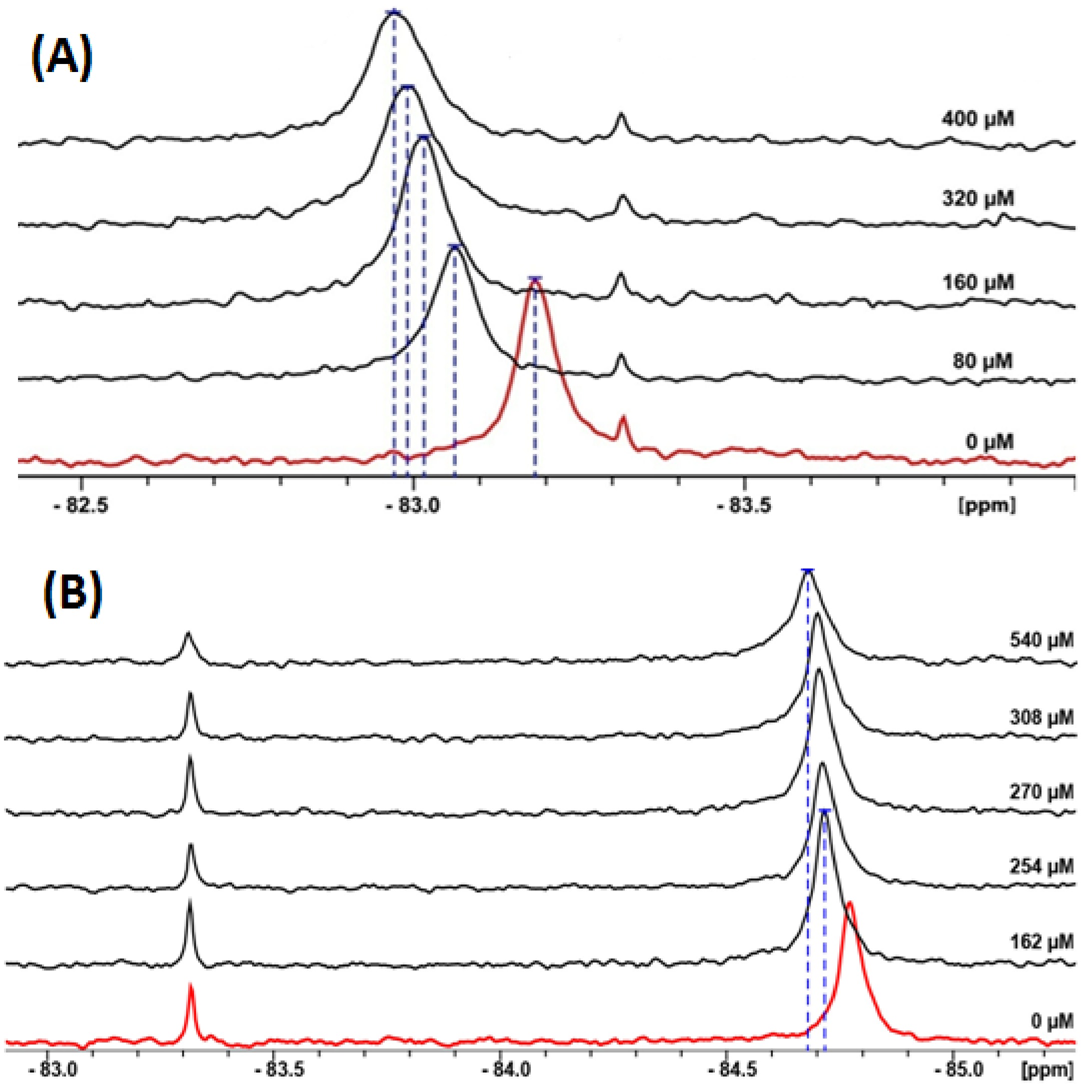

Fig. S38. ^{19}F-NMR spectra of the titration of clavulanic acid into Y58C* SPM-1 (A) or F151C* SPM-1 (B) solutions. The assay mixtures contained 40 µM SPM-1* in 50 mM Tris, pH 7.5, in 90 % H_2O and 10 % D_2O.

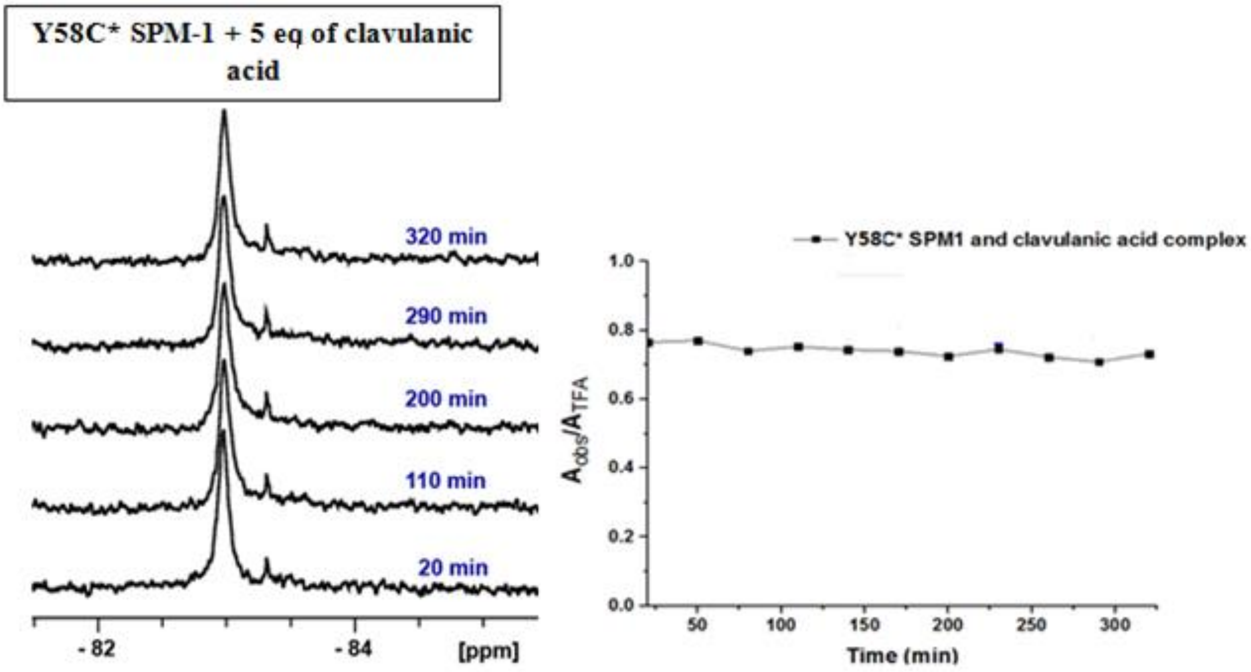

Fig. S39. ^{19}F-NMR time-course spectra of SPM-1* variants with clavulanic acid. Y58C* SPM-1 appears to form (a) stable complex(es) on reaction with sodium clavulanate (or fragments thereof, left). The peak intensity of the complex and its integrative area remained similar over the time course as shown (by comparison to the internal standard, TFA (right). The assay mixture contained 40 µM SPM-1* in 50 mM Tris, pH 7.5, in 90 % H_2O and 10 % D_2O.

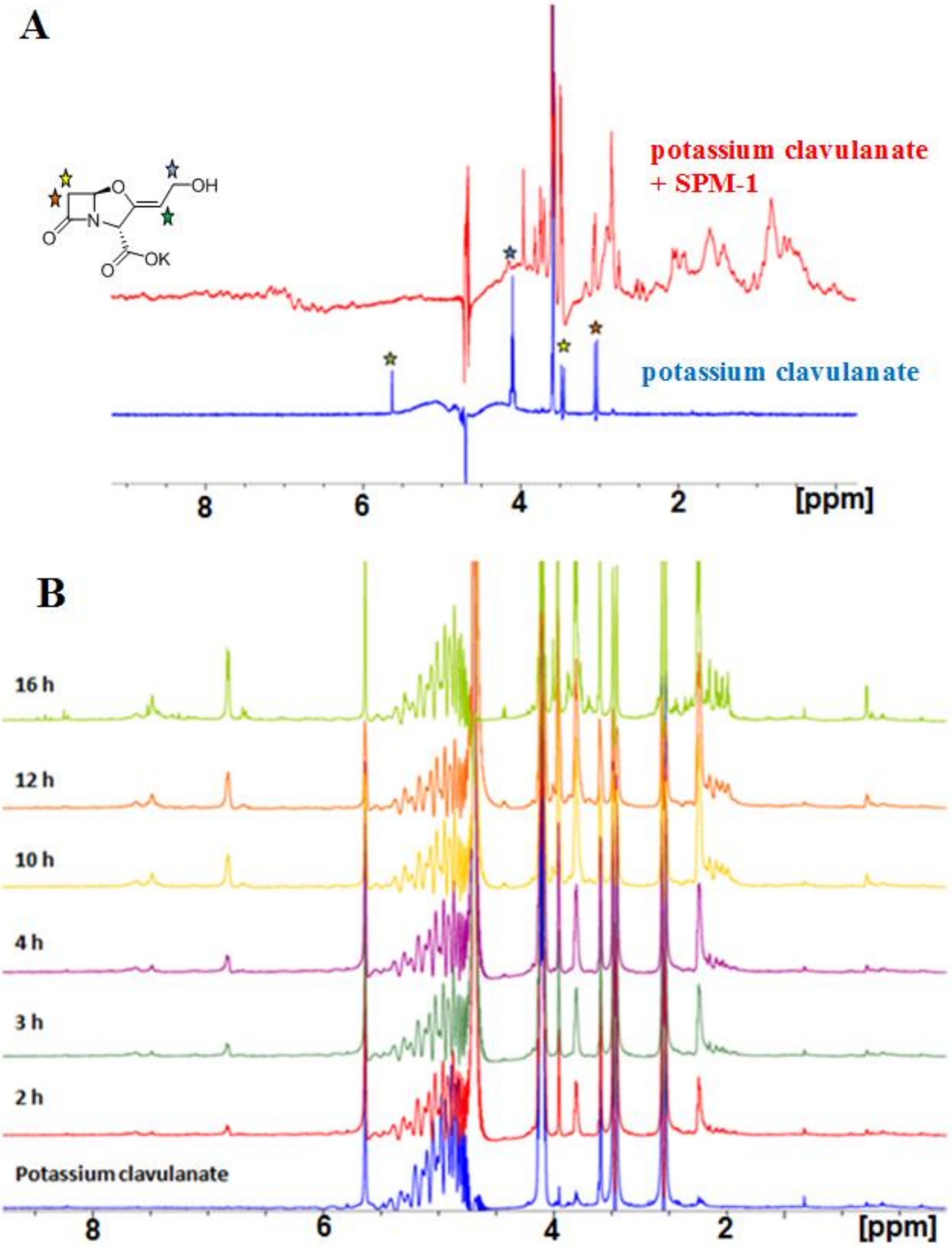

Fig. S40. ^{1}H NMR analyses of the reaction of clavulanic acid with SPM-1. (A) Binding with SPM-1 and **(B)** time course reaction monitoring by ^{1}H NMR. The assay mixture contained 400 µM potassium clavulanate (blue trace), in the presence of 40 µM SPM-1 (red trace) after monitoring the reaction for 10 min in 50 mM Tris-D_{11}, pH 7.5, in 90 % H_2O and 10 % D_2O.

Supplementary Tables

Table S1. Data collection and refinement statistics.

	SPM-1 Y58C
**Data collection**	
Space group	$P4_12_12$
No. of molecules / ASU	2
Cell dimensions	
a, *b*, *c* (Å)	58.89, 59.89 276.11
α, β, γ (°)	90, 90, 90
Wavelength	0.96862
Resolution (Å)	29.26 – 1.75 (1.79 – 1.75)
R_{pim}	0.020 (0.205)
$I / \sigma I$	32.2 (5.1)
$CC_{1/2}$	1.000 (0.895)
Completeness (%)	97.9 (86.8)
Redundancy	25.0 (24.1)
**Refinement**	
Resolution (Å)	29.26 – 1.75
No. reflections	50437
R_{work} / R_{free}	18.40 / 16.59
No. atoms	
Protein	3814
Ligand/ion	5
Water	374
B-factors	
Protein	31.5
Ligand/ion	25.4
Water	42.8
R.m.s. deviations	
Bond lengths (Å)	0.007
Bond angles (°)	1.159
Ramachandran Plot	
Outliers	0.65
Allowed	1.3
Favored (%)	98.06

*Values in parentheses are for highest-resolution shell.

Table S2. Table summarizing the NMR observations with SPM-1.

Note, $\Delta\delta_{max} = \delta_{max} - \delta_0$ represents the chemical shift difference (in ppm) between δ_{max} and δ_0, in which $\delta_{max} = \delta$ observed in the presence of 400 μM of the ligand and $\delta_0 = \delta$ of the SPM-1* variant in the absence of added ligand: δ_0 = -83.15 ppm for Y58C* SPM-1 and δ_0 = -84.75 ppm for F151C* SPM-1 (**Fig. S18**) (reference: trifluoroacetic acid, TFA, δ_{TFA} = -75.45 ppm). For $\Delta\delta_{max}$ < 0.1 ppm, which was used as a cut-off limit, observations were denoted as 'no substantial changes'. The compounds were stable over the course of the experiments, except when undergoing hydrolysis. Potassium clavulanate and tazobactam fragment in solution. Assignment of slow/fast exchanges and of peaks corresponding to protein.inhibitor complexes should be regarded as probable and relative to the chemical shift time scale.

	**Summary of PrOF NMR analyses of SPM-1***				**Other Observations**
	**Ligand titration analyses**		**Time-course monitoring (12 h)**		
	**Y58C* SPM-1 (L3 variant)**	**F151C* SPM-1 (α3 variant)**	**Y58C* SPM-1 (L3 variant)**	**F151C* SPM-1 (α3 variant)**	
**1,10-*o*-Phenanthroline** 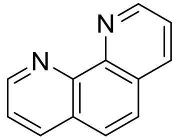	Signal reduction on increased concentrations of the ligand and appearance of a new peak	• Appearance of a new peak<br>• $\Delta\delta_{max}$ = 0.3 ppm	Stable protein-inhibitor peak at -83.8 ppm	Stable protein-inhibitor peak at -84.45 ppm	• Binding of 1,10-*o*-phenanthroline to SPM-1 was studied using ^{1}H CPMG.<br>• The fluorine peaks observed in the presence of 1,10-*o*-phenanthroline are consistent with *apo*-SPM-1* peaks.<br>• 1,10-*o*-phenanthroline does not induce changes in *apo*-Y58C* SPM-1 spectrum.

**ML302** 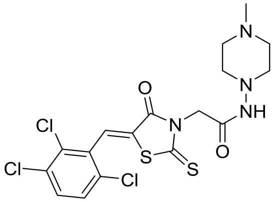	• Appearance of a new peak<br>• Chemical shift, $\Delta\delta_{max}$ = 0.6 ppm<br>• Slow-exchange	• Appearance of a new peak<br>• Chemical shift, $\Delta\delta_{max}$ = 0.35 ppm<br>• Slow-exchange	Stable protein-inhibitor peak observed at -83.75 ppm	Stable protein-inhibitor peak observed at -84.4 ppm	Binding of ML302 to SPM-1 was monitored (^{1}H CPMG).<br>Note ML302 is hydrolysed to ML302F.
**ML302F** 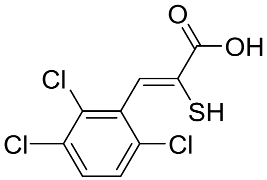	• Appearance of a new peak<br>• Chemical shift, $\Delta\delta_{max}$ = 0.6 ppm<br>• Slow-exchange	• Appearance of a new peak<br>• Chemical shift, $\Delta\delta_{max}$ = 0.35 ppm<br>• Slow-exchange	Stable protein-inhibitor peak observed at -83.75 ppm	Stable protein-inhibitor peak observed at -84.4 ppm	Binding of ML302F to SPM-1 was monitored (^{1}H CPMG).
**L-Captopril** 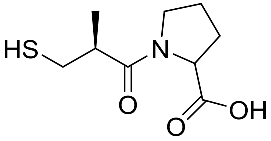	No substantial changes	No substantial changes	No substantial changes	No substantial changes	No/very weak binding of L-captopril to SPM-1 was observed by ^{1}H CPMG.

**Isoquinoline derivative (1)**<br>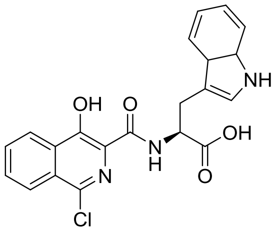	Significant line broadening and disappearance of the signal within the baseline (even at low ligand concentrations)	• Line broadening<br>• $\Delta\delta_{max}$ = 0.75 ppm<br>• Fast/intermediate-exchange	Very broad peak close to the baseline	Stable protein-inhibitor peak at -84 ppm	• Binding of the isoquinoline **(1)** to SPM-1 was studied using ^{1}H CPMG.<br>• Addition of ML302F to Y58C* SPM-1 led to some displacement of isoquinoline **(1)** with the ML302F.Y58C* SPM-1 complex peak appearing and a new peak observed ($\Delta\delta_{max}$ = 0.95 ppm).<br>• Isoquinoline **(1)** was observed to bind to *apo*-Y58C* SPM-1.
**Avibactam**<br>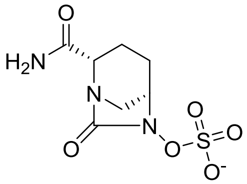	• Line broadening<br>• $\Delta\delta_{max}$ = 0.1 ppm<br>• Fast-exchange	No substantial changes	The peak shifts back towards original protein peak position	No substantial changes	• Weak binding of avibactam to SPM-1 and slow hydrolysis were monitored (CPMG).[22]<br>• Hydrolyzed products do not appear to bind to SPM-1 (wLOGSY).[22]<br>• Addition of intact avibactam after 24 h led to shifting the equilibrium back to the avibactam.Y58C* SPM-1 complex peak.

**Meropenem**<br>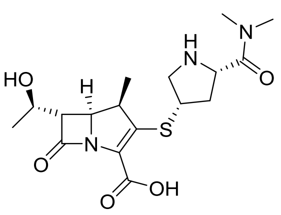	• Line broadening<br>• $\Delta\delta_{max}$ = 0.20 ppm<br>• Fast-exchange	No substantial changes	Stable protein-product peak observed at -82.95 ppm	No substantial changes	• Binding of meropenem to SPM-1 and its hydrolysis were monitored by ^{1}H NMR.<br>• Meropenem was hydrolyzed by BcII and its product was observed to bind to SPM-1 (wLOGSY and PrOF).
**Piperacillin**<br>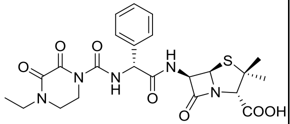	• Line broadening<br>• $\Delta\delta_{max}$ = 0.40 ppm<br>• Fast/intermediate-exchange	No substantial changes	Additional downfield shift ($\Delta\delta$ = 0.18 ppm) and line broadening	No substantial changes	• Binding/hydrolysis of piperacillin with SPM-1 reveals time-dependent (5*R*)-PA epimerisation of the product to (5*S*)-PA.<br>• Piperacillin was hydrolyzed by BcII and PA products were observed to bind to SPM-1 (^{1}H, wLOGSY and PrOF).
**Tazobactam**<br>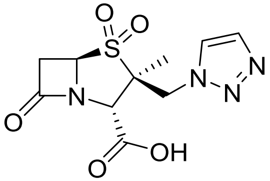	• Line broadening<br>• $\Delta\delta_{max}$ = 0.13 ppm<br>• Fast-exchange	No substantial changes	Stable protein-product peak observed at -83.02 ppm	No substantial changes	Binding of tazobactam to wt SPM-1 and tazobactam hydrolysis/fragmentation were observed by ^{1}H NMR.

**Potassium clavulanate** 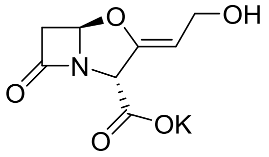	• Line broadening<br>• $\Delta\delta_{max}$ = 0.17 ppm<br>• Fast-exchange	No substantial changes	Stable protein-product peak observed at -82.98 ppm	No substantial changes	Binding of potassium clavulanate/clavulanate fragments to wt SPM-1 and clavulanate hydrolysis/fragmentation were observed by ^{1}H NMR.

Table S3. Table summarizing the K_D values of the ligands with SPM-1* as observed by PrOF NMR.

Compound	K_D (μM)
**ML302**	43 ± 5*
**ML302F**	57 ± 6*
**L-Captopril**	> 400**
**Avibactam**	> 400**
**Meropenem product**	100 ± 10
**Piperacillin products**	275 ± 17

K_D values were fitted using the following equation:[2]

$$\Delta\delta_{obs} = \Delta\delta_{max}\left\{([P]_t + [L]_t + K_d) - [([P]_t + [L]_t + K_d)^2 - 4[P]_t[L]_t]^{1/2}\right\}/2[P]_t$$

$\Delta\delta_{max} = \delta_{max} - \delta_0$ represents the chemical shift difference (in ppm) between δ_{max} and δ_0, in which $\delta_{max} = \delta$ observed in the presence of 400 μM of the ligand and $\delta_0 = \delta$ of SPM-1* variant in the absence of added ligand; $\Delta\delta_{obs} = \delta_{obs} - \delta_0$ represents the chemical shift difference (in ppm) between δ_{obs} and δ_0, in which $\delta_{obs} = \delta$ observed at an added ligand concentration; $[P]_t$ is the total protein concentration; $[L]_t$ is the total ligand concentration.

* For slow-exchange systems, the changes in the integration of the resonance corresponding to the protein-ligand complex were fitted instead of the chemical shift changes.

** For very weak binders, an estimate of the K_D was given as the observed curves did not reach saturation.

*** K_D was not determined for 1,10-*o*-phenanthroline or **(1)**. The behavior of 1,10-*o*-phenanthroline does not follow standard kinetics (likely due to the competition involving the binding of the metal to the protein and that of the metal to 1,10-*o*-phenanthroline). Addition of **(1)** led to very substantial signal broadening such that it merged with the baseline.

**** Values are not given for clavulanate and tazobactam since these serine β-lactamase inhibitors undergo hydrolysis to multiple products.[23]

References

[1] A. Makena, S. S. van Berkel, C. Lejeune, R. J. Owens, A. Verma, R. Salimraj, J. Spencer, J. Brem, C. J. Schofield, *ChemMedChem* **2013**, *8*, 1923-1929.

[2] A. M. Rydzik, J. Brem, S. S. van Berkel, I. Pfeffer, A. Makena, T. D. Claridge, C. J. Schofield, *Angew. Chem.***2014**, *53*, 3129-3133.

[3] J. Brem, W. B. Struwe, A. M. Rydzik, H. Tarhonskaya, I. Pfeffer, E. Flashman, S. S. van Berkel, J. Spencer, T. D. Claridge, M. A. McDonough, J. L. Benesch, C. J. Schofield, *Chem. Sci.* **2015**, *6*, 956-963.

[4] a) A. Makena, A. O. Duzgun, J. Brem, M. A. McDonough, A. M. Rydzik, M. I. Abboud, A. Saral, A. C. Cicek, C. Sandalli, C. J. Schofield, *Antimicrob. Agents Chemother.* **2016**, *60*, 1377-1384; b) A. Makena, J. Brem, I. Pfeffer, R. E. Geffen, S. E. Wilkins, H. Tarhonskaya, E. Flashman, L. M. Phee, D. W. Wareham, C. J. Schofield, *J. Antimicrob. Chemother.* **2015**, *70*, 463-469.

[5] W. Kabsch, *Acta Crystallogr. D* **2010**, *66*, 125-132.

[6] M. D. Winn, C. C. Ballard, K. D. Cowtan, E. J. Dodson, P. Emsley, P. R. Evans, R. M. Keegan, E. B. Krissinel, A. G. W. Leslie, A. McCoy, S. J. McNicholas, G. N. Murshudov, N. S. Pannu, E. A. Potterton, H. R. Powell, R. J. Read, A. Vagin, K. S. Wilson, *Acta Crystallogr. D: Biol. Crystallogr.* **2011**, *67*, 235-242.

[7] A. J. McCoy, R. W. Grosse-Kunstleve, P. D. Adams, M. D. Winn, L. C. Storoni, R. J. Read, *J. Appl.Crystallogr.* **2007**, *40*, 658-674.

[8] P. D. Adams, P. V. Afonine, G. Bunkoczi, V. B. Chen, I. W. Davis, N. Echols, J. J. Headd, L.-W. Hung, G. J. Kapral, R. W. Grosse-Kunstleve, A. J. McCoy, N. W. Moriarty, R. Oeffner, R. J. Read, D. C. Richardson, J. S. Richardson, T. C. Terwilliger, P. H. Zwart, *Acta Crystallogr. D* **2010**, *66*, 213-221.

[9] P. Emsley, K. Cowtan, *Acta Crystallogr. D* **2004**, *60*, 2126-2132.

[10] V. B. Chen, W. B. Arendall, J. J. Headd, D. A. Keedy, R. M. Immormino, G. J. Kapral, L. W. Murray, J. S. Richardson, D. C. Richardson, *Acta Crystallogr. D* **2010**, *66*, 12-21.

[11] S. S. van Berkel, J. Brem, A. M. Rydzik, R. Salimraj, R. Cain, A. Verma, R. J. Owens, C. W. Fishwick, J. Spencer, C. J. Schofield, *J. Med. Chem.* **2013**, *56*, 6945-6953.

[12] J. Brem, S. S. van Berkel, W. Aik, A. M. Rydzik, M. B. Avison, I. Pettinati, K. D. Umland, A. Kawamura, J. Spencer, T. D. Claridge, M. A. McDonough, C. J. Schofield, *Nat. Chem.* **2014**, *6*, 1084-1090.

[13] J. A. Aguilar, M. Nilsson, G. Bodenhausen, G. A. Morris, *Chem. Commun.* **2012**, *48*, 811-813.

[14] C. Dalvit, P. Pevarello, M. Tato, M. Veronesi, A. Vulpetti, M. Sundstrom, *J. Biomol.NMR* **2000**, *18*, 65-68.

[15] J. H. Toney, G. G. Hammond, P. M. Fitzgerald, N. Sharma, J. M. Balkovec, G. P. Rouen, S. H. Olson, M. L. Hammond, M. L. Greenlee, Y. D. Gao, *J. Biol. Chem.* **2001**, *276*, 31913-31918.

[16] G. Garau, C. Bebrone, C. Anne, M. Galleni, J. M. Frere, O. Dideberg, *J. Mol. Biol.* **2005**, *345*, 785-795.

[17] M. R. Meini, L. I. Llarrull, A. J. Vila, *Antibiotics* **2014**, *3*, 285-316.

[18] J. Brem, W. B. Struwe, A. M. Rydzik, H. Tarhonskaya, I. Pfeffer, E. Flashman, S. S. van Berkel, J. Spencer, T. D. Claridge, M. A. McDonough, J. L. Benesch, C. J. Schofield, *Chem. Sci.* **2015**, *6*, 956-963.

[19] J. Brem, S. S. van Berkel, D. Zollman, S. Y. Lee, O. Gileadi, P. J. McHugh, T. R. Walsh, M. A. McDonough, C. J. Schofield, *Antimicrob. Agents Chemother.* **2016**, *60*, 142-150.

[20] D. T. King, L. J. Worrall, R. Gruninger, N. C. Strynadka, *J. Am. Chem. Soc.* **2012**, *134*, 11362-11365.

[21] S. S. van Berkel, J. E. Nettleship, I. K. Leung, J. Brem, H. Choi, D. I. Stuart, T. D. Claridge, M. A. McDonough, R. J. Owens, J. Ren, C. J. Schofield, *ACS Chem. Biol.* **2013**, *8*, 2112-2116.

[22] M. I. Abboud, C. Damblon, J. Brem, N. Smargiasso, P. Mercuri, B. Gilbert, A. M. Rydzik, T. D. Claridge, C. J. Schofield, J. M. Frere, *Antimicrob. Agents Chemother.* **2016**, *60*, 5655-62.

[23] Z. Liang, L. Li, Y. Wang, L. Chen, X. Kong, Y. Hong, L. Lan, M. Zheng, C. Guang-Yang, H. Liu, X. Shen, C. Luo, K. K. Li, K. Chen, H. Jiang, *PloS One* **2011**, 6, e23606.
